# Modified *Z*-algorithms for reckoning fixed points with application to nonlinear integral equations

**DOI:** 10.1371/journal.pone.0346021

**Published:** 2026-06-03

**Authors:** Habib ur Rehman, Hasanen A. Hammad, Manal Elzain Mohamed Abdalla

**Affiliations:** 1 School of Mathematics, Yunnan Normal University, Kunming, China; 2 Department of Mathematics, College of Sciences, Qassim University, Buraydah, Saudi Arabia; 3 Department of Mathematics, Faculty of Science, Sohag University, Sohag, Egypt; 4 Department of Mathematics, Faculty of Science, King Khalid University‌‌, Abha, Saudi Arabia; Srinath University, INDIA

## Abstract

In this paper, we investigate the existence of common fixed points for C−α nonexpansive mappings. We propose a novel four-step iterative scheme, referred to as the *Z*-iteration, which is specifically developed for handling pairs of such mappings. Using this algorithm, we establish several weak and strong convergence results that guarantee the existence of common fixed points. To substantiate the theoretical results, we present constructive examples. Moreover, the practical utility of the proposed method is demonstrated by applying it to approximate solutions of a specific class of nonlinear integral equations in Banach spaces, with a representative example provided to validate its effectiveness.

## 1 Introduction and preliminaries

The theory concerning the existence of fixed points (FPs) plays a central role in mathematical analysis, particularly in establishing the existence of solutions to equations or transformations without necessarily providing a means of constructing them. A foundational result in this area is the Banach FP theorem, also known as the contraction mapping (CAM) principle, which ensures both the existence and uniqueness of a FP for a contraction mapping in a complete metric space and offers an iterative procedure for its approximation [1,2].

Beyond contractive mappings, other landmark results include the Brouwer FP theorem for continuous self-maps on compact and convex subsets of Euclidean space and its generalization to infinite-dimensional Banach spaces (BSs) via the Schauder FP theorem [2,3]. These theorems are particularly valuable in settings where uniqueness is not guaranteed, but existence is still of interest. In addition, the topological degree theory, an extension of the classical winding number, provides a powerful framework for estimating the number of solutions to nonlinear equations. This theory is closely connected to fixed point analysis and serves as a crucial tool in proving the existence of solutions for various classes of analytical and differential equations [4]. Collectively, these foundational results support a wide range of applications across differential equations, integral equations, and optimization by establishing the theoretical groundwork for solution existence.

The existence theory for nonexpansive mappings was independently initiated by Browder [5], Göhde [6], and Kirk [7]. Their pioneering contributions have since inspired substantial research interest, leading to numerous developments, extensions, and generalizations of this fundamental concept. For an in-depth overview of such advancements, the reader is referred to [8–13].

Let Ω be a nonempty subset of a BS (Ξ,‖·‖). For all ω,υ∈Ξ, a mapping ℜ:Ω→Ω is said to be:

(i) a *contraction* if
$$\begin{array}{c} \|ℜ\omega -ℜ\upsilon \|\leq \varsigma \|\omega -\upsilon \|,\;\text{for some }\varsigma \in [0,1); \end{array}$$
(ii) *nonexpansive* (NE) if
$$\begin{array}{c} \|ℜ\omega -ℜ\upsilon \|\leq \|\omega -\upsilon \|; \end{array}$$
(iii) *quasi-nonexpansive* (quasi-NE) if Fix(ℜ)≠∅ and
$$\begin{array}{c} \|ℜ\omega -\eta \|\leq \|\omega -\eta \|,\;\text{for all }\eta \in \mathrm{Fix}(ℜ), \end{array}$$
  where Fix(ℜ) denotes the set of FPs of ℜ.

It is worth noting that any NE mapping with a FP is necessarily quasi-NE. Building upon this observation, Suzuki [14] introduced a broader class of NE mappings that satisfy a relaxed condition, referred to as *Condition (C)*. A self-mapping ℜ on Ω is said to be a *Suzuki generalized NE mapping* (i.e., ℜ satisfies Condition (C)) if


$$\begin{array}{c} \frac{1}{2}\|\omega -ℜ\omega \|\leq \|\omega -\upsilon \|\;⟹\;\|ℜ\omega -ℜ\upsilon \|\leq \|\omega -\upsilon \|, \end{array}$$

holds for all ω,υ∈Ξ.

Following these developments, Aoyama and Kohsaka [15] introduced the notion of α-NE mappings, representing a significant extension of the existing framework. A self-mapping ℜ on Ω is called α*-NE* if


$$\begin{array}{c} \|ℜ\omega -ℜ\upsilon {\|}^{2}\leq \alpha \|ℜ\omega -\upsilon {\|}^{2}+\alpha \|ℜ\upsilon -\omega {\|}^{2}+(1-2\alpha )\|\omega -\upsilon {\|}^{2} \end{array}$$

for all ω,υ∈Ξ, with α∈[0,1). Clearly, every NE mapping is an 0-NE mapping. It is also important to observe that while NE mappings are inherently continuous, the generalized NE mappings introduced by Suzuki and the α-NE mappings may not be continuous in general.

More recently, Pant and Shukla [16] proposed a further generalization, termed *generalized* α*-NE mappings*. A self-mapping ℜ on Ω is said to be *generalized* α*-NE* if


$$\begin{array}{c} \frac{1}{2}\|\omega -ℜ\omega \|\leq \|\omega -\upsilon \|\;⟹\;\|ℜ\omega -ℜ\upsilon \|\leq \alpha \|ℜ\omega -\upsilon \|+\alpha \|ℜ\upsilon -\omega \|+(1-2\alpha )\|\omega -\upsilon \| \end{array}$$

holds for all ω,υ∈Ξ, with α∈[0,1). The class introduced in [16] does not fully contain the α-NE mappings. To address this gap, the same authors later introduced a more inclusive class known as *C-*α*-NE mappings* [17], which properly encompasses the α-NE class.

We now recall the definition of a *C*-α-NE mapping as follows:

**Definition 1 ([17]).** *Assume that* Ω *is a nonempty subset of a Banach space* Ξ*. A self-mapping* ℜ *on* Ω *is called a C-*α*-NE if the following implication holds:*


$$\begin{array}{c} \frac{1}{2}\|\omega -ℜ\omega \|\leq \|\omega -\upsilon \|\;⟹\;\|ℜ\omega -ℜ\upsilon {\|}^{2}\leq \alpha \|ℜ\omega -\upsilon {\|}^{2}+\alpha \|ℜ\upsilon -\omega {\|}^{2}+(1-2\alpha )\|\omega -\upsilon {\|}^{2}, \end{array}$$ (1)

*for all* ω,υ∈Ξ *and* α∈[0,1).

Since the basic Picard iteration $\omega_{n+1}=ℜ\omega_{n}$ often fails to converge to a FP when applied to NE mappings, a wide variety of more advanced iterative schemes have been proposed. These methods aim to more effectively approximate FPs or common FPs for various classes of NE mappings. Among the most notable iterations, particularly for their balance between simplicity and convergence efficiency are those developed by Ishikawa [18], Agarwal et al. [19], Abbas and Nazir [20], Noor [21], and Ali et al. [22]. For further examples of widely adopted and well-studied iterative methods, the reader is referred to [23–28].

We now recall the concept of the Opial property, which plays an important role in convergence analysis.

**Definition 2 ([29]).** *A BS* Ξ *is said to possess the* Opial property *if, for every weakly convergent sequence* $\left\{\zeta_{n}\right\}$ *in* Ξ *with weak limit* ζ*, the inequality*


$$\begin{array}{c} \underset{n\to \infty }{lim inf}\|\zeta_{n}-\eta \|>\underset{n\to \infty }{lim inf}\|\zeta_{n}-\zeta \| \end{array}$$

*holds for all* η∈Ξ *such that* η≠ζ.

The following lemma will be instrumental in our subsequent analysis.

**Lemma 1 ([30]).** *Let* Ξ *be a uniformly convex BS, and let* $\left\{\phi_{n}\right\}$ *be a sequence satisfying* $0<k\leq \phi_{n}\leq n<1$ *for all* n∈ℕ*. Suppose that* $\left\{\gamma_{n}\right\}$ *and* $\left\{\zeta_{n}\right\}$ *are two sequences in* Ξ *such that*


$$\begin{array}{c} \underset{n\to \infty }{lim sup}\|\gamma_{n}\|\leq \sigma ,\;\underset{n\to \infty }{lim sup}\|\zeta_{n}\|\leq \sigma , \end{array}$$

*and*


$$\begin{array}{c} \lim_{n\to \infty }\left\|\phi_{n}\gamma_{n}+(1-\phi_{n})\zeta_{n}\right\|=\sigma \end{array}$$

*for some* σ≥0*. Then it follows that*


$$\begin{array}{c} \lim_{n\to \infty }\|\gamma_{n}-\zeta_{n}\|=0. \end{array}$$

**Definition 3 ([31]).** *Let* Ω *be a nonempty subset of a uniformly convex BS* Ξ*, and let* $\{\omega_{n}\}\subset \Xi$*. For any* ω∈Ξ*, define*


$$\begin{array}{c} s\left(\omega ,\{\omega_{n}\}\right):=\underset{n\to \infty }{lim sup}\|\omega_{n}-\omega \|. \end{array}$$

*The asymptotic radius of the sequence* $\left\{\omega_{n}\right\}$ *relative to* Ω *is given by*


$$\begin{array}{c} s\left(\Omega ,\{\omega_{n}\}\right):=\inf\left\{s\left(\omega ,\{\omega_{n}\}\right):\omega \in \Omega \right\}. \end{array}$$

*The asymptotic center of* $\left\{\omega_{n}\right\}$ *relative to* Ω *is defined by*


$$\begin{array}{c} R\left(\Omega ,\{\omega_{n}\}\right):=\left\{\omega \in \Omega :s\left(\omega ,\{\omega_{n}\}\right)=s\left(\Omega ,\{\omega_{n}\}\right)\right\}. \end{array}$$

*It is a standard result that, in a uniformly convex Banach space, the asymptotic center* $R\left(\Omega ,\{\omega_{n}\}\right)$ *is a singleton.*

**Definition 4 ([32]).** *Let* Ω *be a nonempty, bounded, closed, and convex subset of a BS* Ξ*. A sequence* $\left\{\omega_{n}\right\}$ *is said to be an asymptotic center for a mapping* ℜ:Ω→Ξ *if*


$$\begin{array}{c} \underset{n\to \infty }{lim sup}\|ℜ\omega -\omega_{n}\|\leq \underset{n\to \infty }{lim sup}\|\omega_{n}-\omega \|,\;\text{for all }\omega \in \Omega . \end{array}$$

**Definition 5 ([33]).** *Let* $\left\{\theta_{n}\right\}$ *and* $\left\{\hbar_{n}\right\}$ *be two iterative schemes for FP approximation, both converging to the same fixed point* η*. Suppose their respective error estimates satisfy*


$$\begin{array}{c} \|\theta_{n}-\eta \|\leq b_{n}\;\text{and}\;\|\hbar_{n}-\eta \|\leq c_{n}, \end{array}$$

*where* $\left\{b_{n}\right\}$ *and* $\left\{c_{n}\right\}$ *are sequences of positive real numbers converging to zero. If*


$$\begin{array}{c} \lim_{n\to \infty }\frac{b_{n}}{c_{n}}=0, \end{array}$$

*then the sequence* $\left\{\theta_{n}\right\}$ *is said to converge to* η *faster than* $\left\{\hbar_{n}\right\}$.

**Definition 6 ([33]).** *Let* $\left\{\hbar_{n}\right\}$ *and* $\left\{\varphi_{n}\right\}$ *be two sequences generated by iterative algorithms that converge to a common FP* η*. The sequence* $\left\{\hbar_{n}\right\}$ *is said to converge to* η *faster than* $\left\{\varphi_{n}\right\}$ *if*


$$\begin{array}{c} \lim_{n\to \infty }\frac{\left\|\hbar_{n}-\eta \right\|}{\left\|\varphi_{n}-\eta \right\|}=0. \end{array}$$

Modified *Z*-algorithms are effectively utilized in solving physical problems that can be reduced to one-dimensional pattern recognition or sequence analysis. A prominent area is signal processing, where these algorithms can be adapted for faster detection of specific patterns, transients, or anomalies within large time-series data, such as finding a particular waveform signature or seismic signal for diagnosis or event localization. They are also conceptually applied in bioinformatics. The core utility lies in their linear-time complexity for finding all occurrences of a pattern and its longest prefixes that match a prefix of the text, offering a computational efficiency crucial for handling the immense datasets typical of modern physical and biological sensing systems.

Motivated by recent developments and powerful concepts in FP theory, this work investigates the convergence behavior of *C*-α-NE mappings in uniformly convex BSs. To this end, we employ a modified four-step iterative scheme, referred to as the *modified Z-iteration* (abbreviated as MZI), in conjunction with several auxiliary results to analyze convergence properties. Theoretical results are supported by meaningful numerical examples, including detailed tabular and graphical illustrations. Furthermore, we demonstrate the practical utility of the proposed method by applying it to a specific class of Volterra-Fredholm integral equations (VFIEs), for which we provide approximate solutions.

Our paper is organized as follows: Section 2 is dedicated to an investigation of the convergence rate of the proposed algorithm, achieved through the statement and proof of several auxiliary lemmas and two theorems. In Section 3, we analyze and compare convergence rates, focusing on how the proposed algorithm’s performance measures up against several established iterative methods. In Section 4, we provide examples that illustrate and validate our findings. The numerical performance of the proposed iterative scheme is assessed in Section 5. Section 6 applies the established results to investigate the existence of solutions for a general class of VFIEs with a deviating argument in a BS. The paper concludes in Section 7, where we summarize our findings and suggest promising directions for future research. For reference, a list of abbreviations is provided in Section 8.

## 2 Convergence rate of Z−Iterative methods

Throughout this paper, we denote by ℕ the set of natural numbers and by ℝ the set of real numbers. We begin by stating a fundamental lemma regarding the properties of the set of common FPs.

**Lemma 2.** *Let* Ω *be a nonempty, bounded, closed, and convex subset of a BS* Ξ*. Suppose that* ${ℜ}_{1},{ℜ}_{2},{ℜ}_{3}:\Omega \to \Omega$ *are three C-*α*-NE mappings satisfying condition* (1)*. Then the set*


$$\begin{array}{c} ℵ:=\mathrm{Fix}({ℜ}_{1})\cap \mathrm{Fix}({ℜ}_{2})\cap \mathrm{Fix}({ℜ}_{3}) \end{array}$$

*is nonempty and closed. Moreover, if* Ξ *is strictly convex, then* ℵ *is also convex.*

*Proof.* Consider a sequence $\left\{\nu_{n}\right\}$ in $ℵ=fix\left({ℜ}_{1}\right)\cap fix\left({ℜ}_{2}\right)\cap fix\left({ℜ}_{3}\right)$ converging to ν∈Ω. As $\left\{\nu_{n}\right\}$ is a sequence in both $fix\left({ℜ}_{1}\right),$ $fix\left({ℜ}_{2}\right),$ and $fix\left({ℜ}_{3}\right)$, and acknowledging that ${ℜ}_{1},$ ${ℜ}_{2}$ and ${ℜ}_{3}$ are C−α−NE mappings, it follows that


$$\begin{array}{c} \frac{1}{2}\left\|\nu_{n}-{ℜ}_{1}\nu_{n}\right\|=0\leq \left\|\nu_{n}-\nu \right\|, \end{array}$$

implies for n∈ℕ,


$$\begin{array}{cc} \lim_{n\to \infty }\left\|\nu_{n}-{ℜ}_{1}\nu \right\| & =\lim_{n\to \infty }{\left\|{ℜ}_{1}\nu_{n}-{ℜ}_{1}\nu \right\|}^{2}\\ & \leq \lim_{n\to \infty }\left[\alpha {\left\|{ℜ}_{1}\nu_{n}-\nu \right\|}^{2}+\alpha {\left\|\nu_{n}-{ℜ}_{1}\nu \right\|}^{2}+(1-2\alpha ){\left\|\nu_{n}-\nu \right\|}^{2}\right]\\ & =\lim_{n\to \infty }\left[\alpha {\left\|\nu_{n}-\nu \right\|}^{2}+\alpha {\left\|\nu_{n}-{ℜ}_{1}\nu \right\|}^{2}+(1-2\alpha ){\left\|\nu_{n}-\nu \right\|}^{2}\right]. \end{array}$$

Hence,


$$\begin{array}{c} \left(1-\alpha \right)\lim_{n\to \infty }\left\|\nu_{n}-{ℜ}_{1}\nu \right\|\leq \lim_{n\to \infty }\left[(1-\alpha ){\left\|\nu_{n}-\nu \right\|}^{2}\right]. \end{array}$$

Since 1−α>0, one has


$$\begin{array}{c} \lim_{n\to \infty }\left\|\nu_{n}-{ℜ}_{1}\nu \right\|\leq \lim_{n\to \infty }{\left\|\nu_{n}-\nu \right\|}^{2}=0, \end{array}$$

which implies that $\left\{\nu_{n}\right\}$ converges to ${ℜ}_{1}\nu ,$ that is, $\nu ={ℜ}_{1}\nu$ and $\nu \in fix\left({ℜ}_{1}\right).$

By following a similar procedure, we establish that $\nu \in fix\left({ℜ}_{2}\right)$ and $\nu \in fix\left({ℜ}_{3}\right)$. This implies that ν∈ℵ, which in turn demonstrates that ℵ is closed.

Next, consider the case where Ξ is strictly convex and Ω is convex. Fix λ∈(0,1) and ω,τ∈ℵ such that ω≠τ and set ν=γω+(1−γ)τ∈Ω. Given that ${ℜ}_{1},$ ${ℜ}_{2}$ and ${ℜ}_{3}$ are C−α−NE mappings, we begin by observing


$$\begin{array}{c} \frac{1}{2}\left\|\omega -{ℜ}_{1}\omega \right\|=0\leq \left\|\omega -\nu \right\|, \end{array}$$

and applying (1), one can write


$$\begin{array}{cc} {\left\|{ℜ}_{1}\omega -{ℜ}_{1}\nu \right\|}^{2} & \leq \alpha {\left\|{ℜ}_{1}\omega -\nu \right\|}^{2}+\alpha {\left\|\omega -{ℜ}_{1}\nu \right\|}^{2}+(1-2\alpha ){\left\|\omega -\nu \right\|}^{2}\\ & =\alpha {\left\|\omega -\nu \right\|}^{2}+\alpha {\left\|\omega -{ℜ}_{1}\nu \right\|}^{2}+(1-2\alpha ){\left\|\omega -\nu \right\|}^{2}\\ & =\alpha {\left\|\omega -{ℜ}_{1}\nu \right\|}^{2}+(1-\alpha ){\left\|\omega -\nu \right\|}^{2}\\ & =\alpha {\left\|{ℜ}_{1}\omega -{ℜ}_{1}\nu \right\|}^{2}+(1-\alpha ){\left\|\omega -\nu \right\|}^{2}, \end{array}$$

it follows that


$$\begin{array}{c} (1-\alpha ){\left\|{ℜ}_{1}\omega -{ℜ}_{1}\nu \right\|}^{2}\leq (1-\alpha ){\left\|\omega -\nu \right\|}^{2}. \end{array}$$

Since 1−α>0, one has


$$\begin{array}{c} {\left\|{ℜ}_{1}\omega -{ℜ}_{1}\nu \right\|}^{2}\leq {\left\|\omega -\nu \right\|}^{2}⟹\left\|{ℜ}_{1}\omega -{ℜ}_{1}\nu \right\|\leq \left\|\omega -\nu \right\|. \end{array}$$

Additionally,


$$\begin{array}{cc} \nu & =\gamma \omega +(1-\gamma )\tau \\ & =\gamma \omega +(1-\gamma )\tau +(1-\gamma )\omega -(1-\gamma )\omega \\ & =-(1-\gamma )\left(\omega -\tau \right)+\omega . \end{array}$$

Hence,


$$\begin{array}{c} \omega -\nu =(1-\gamma )\left(\omega -\tau \right). \end{array}$$

The norm of both sides of the expression above leads to


$$\begin{array}{c} \left\|\omega -\nu \right\|=(1-\gamma )\left\|\omega -\tau \right\|. \end{array}$$ (2)

Analogously, for τ∈ℵ, one has


$$\begin{array}{c} {\left\|{ℜ}_{1}\tau -{ℜ}_{1}\nu \right\|}^{2}\leq {\left\|\tau -\nu \right\|}^{2}⟹\left\|{ℜ}_{1}\tau -{ℜ}_{1}\nu \right\|\leq \left\|\tau -\nu \right\|. \end{array}$$ (3)

Since Ξ is strictly convex, there is ς∈(0,1) such that


$$\begin{array}{cc} {ℜ}_{1}\nu & =\varsigma \omega +(1-\varsigma )\tau \\ & =\varsigma \omega +(1-\varsigma )\tau +(1-\varsigma )\omega -(1-\varsigma )\omega \\ & =-(1-\varsigma )\left(\omega -\tau \right)+\omega , \end{array}$$

which implies that


$$\begin{array}{c} \left(\omega -{ℜ}_{1}\nu \right)=\left({ℜ}_{1}\omega -{ℜ}_{1}\nu \right)=(1-\varsigma )\left(\omega -\tau \right). \end{array}$$

The norm of both sides of the expression above leads to


$$\begin{array}{c} \left\|{ℜ}_{1}\omega -{ℜ}_{1}\nu \right\|=(1-\varsigma )\left\|\omega -\tau \right\|. \end{array}$$ (4)

From (2) in (4), we get


$$\begin{array}{c} (1-\varsigma )\left\|\omega -\tau \right\|=\left\|{ℜ}_{1}\omega -{ℜ}_{1}\nu \right\|\leq \left\|\omega -\nu \right\|=(1-\gamma )\left\|\omega -\tau \right\|. \end{array}$$ (5)

A similar calculation, incorporating (3), yields:


$$\begin{array}{c} \varsigma \left\|\omega -\tau \right\|=\left\|{ℜ}_{1}\tau -{ℜ}_{1}\nu \right\|\leq \left\|\tau -\nu \right\|=\gamma \left\|\omega -\tau \right\|. \end{array}$$ (6)

It follows from (5) and (6) that (1−ς)≤(1−γ) and ς≤γ. This is valid only if ς=γ. Therefore, $\nu \in fix\left({ℜ}_{1}\right).$ Through analogous calculations, it can further be demonstrated that $\nu \in fix\left({ℜ}_{2}\right)$ and $\nu \in fix\left({ℜ}_{3}\right)$. This leads to the conclusion that ν∈ℵ, establishing the convexity of ℵ. □

Further in this section, we establish convergence results for a pair of *C*-α-NE mappings. Before proceeding, we introduce a novel four-step iterative algorithm involving three mappings ${ℜ}_{1},{ℜ}_{2},{ℜ}_{3}:\Omega \to \Omega$, where Ω is a nonempty subset of a BS Ξ. The algorithm is defined as follows:


$$\begin{array}{c} \left\{ \begin{array}{cc} & \omega_{0}\in \Omega ,\\ & \nu_{n}=(1-\rho_{n})\tau_{n}+\rho_{n}{ℜ}_{1}\tau_{n},\\ & \tau_{n}=(1-\xi_{n})\kappa_{n}+\xi_{n}{ℜ}_{2}\kappa_{n},\\ & \kappa_{n}={ℜ}_{3}\left[(1-\lambda_{n})\omega_{n}+\lambda_{n}{ℜ}_{3}\omega_{n}\right],\\ & \omega_{n+1}={ℜ}_{3}\nu_{n}, \end{array}\right. \end{array}$$ (7)

for all n=0,1,2,…, where the sequences $\left\{\rho_{n}\right\}$, $\left\{\xi_{n}\right\}$, and $\left\{\lambda_{n}\right\}$ are chosen from the interval (0,1). We refer to this iterative process as the MZI. In the sequel, we present a fundamental result associated with this method.

**Lemma 3.** *Let* Ω *be a nonempty, bounded, closed, and convex subset of a Banach space* Ξ*. Suppose that* ${ℜ}_{1},{ℜ}_{2},{ℜ}_{3}:\Omega \to \Omega$ *are three C-*α*-NE mappings with a nonempty common FP set*


$$\begin{array}{c} ℵ:=\mathrm{Fix}({ℜ}_{1})\cap \mathrm{Fix}({ℜ}_{2})\cap \mathrm{Fix}({ℜ}_{3}). \end{array}$$

*If* η∈ℵ *and* $\left\{\omega_{n}\right\}$ *is the sequence generated by the MZI scheme (7), then the limit*


$$\begin{array}{c} \lim_{n\to \infty }\|\omega_{n}-\eta \| \end{array}$$

*exists for every* η∈ℵ.

*Proof.* Since ${ℜ}_{1},{ℜ}_{2},{ℜ}_{3}:\Omega \to \Omega$ are three C−α−NE mappings, one has


$$\begin{array}{c} \left\|{ℜ}_{1}\omega -\eta \right\|\leq \left\|\omega -\eta \right\|,\;\left\|{ℜ}_{2}\omega -\eta \right\|\leq \left\|\omega -\eta \right\|,\text{ and }\left\|{ℜ}_{3}\omega -\eta \right\|\leq \left\|\omega -\eta \right\|, \end{array}$$

for each η∈ℵ. As ${ℜ}_{1},{ℜ}_{2}$ and ${ℜ}_{3}$ are quasi-NE maps, then by the algorithm (7), we have


$$\begin{array}{cc} \left\|\omega_{n+1}-\eta \right\| & =\left\|{ℜ}_{3}\nu_{n}-\eta \right\|\\ & \leq \left\|\nu_{n}-\eta \right\|\\ & =\left\|\left(1-\rho_{n}\right)\tau_{n}+\rho_{n}{ℜ}_{1}\tau_{n}-\eta \right\|\\ & =\left\|\left(1-\rho_{n}\right)\left(\tau_{n}-\eta \right)+\rho_{n}\left({ℜ}_{1}\tau_{n}-\eta \right)\right\|\\ & \leq \left(1-\rho_{n}\right)\left\|\tau_{n}-\eta \right\|+\rho_{n}\left\|{ℜ}_{1}\tau_{n}-\eta \right\|\\ & \leq \left(1-\rho_{n}\right)\left\|\tau_{n}-\eta \right\|+\rho_{n}\left\|\tau_{n}-\eta \right\|\\ & =\left\|\tau_{n}-\eta \right\|\\ & =\left\|\left(1-\xi_{n}\right)\kappa_{n}+\xi_{n}{ℜ}_{2}\kappa_{n}-\eta \right\|\\ & \leq \left(1-\xi_{n}\right)\left\|\kappa_{n}-\eta \right\|+\xi_{n}\left\|{ℜ}_{2}\kappa_{n}-\eta \right\|\\ & \leq \left(1-\xi_{n}\right)\left\|\kappa_{n}-\eta \right\|+\xi_{n}\left\|\kappa_{n}-\eta \right\|\\ & =\left\|\kappa_{n}-\eta \right\|\\ & =\left\|{ℜ}_{3}\left[\left(1-\lambda_{n}\right)\omega_{n}+\lambda_{n}{ℜ}_{3}\omega_{n}\right]-\eta \right\|\\ & \leq \left\|\left(1-\lambda_{n}\right)\omega_{n}+\lambda_{n}{ℜ}_{3}\omega_{n}-\eta \right\|\\ & \leq \left(1-\lambda_{n}\right)\left\|\omega_{n}-\eta \right\|+\lambda_{n}\left\|{ℜ}_{3}\omega_{n}-\eta \right\|\\ & \leq \left(1-\lambda_{n}\right)\left\|\omega_{n}-\eta \right\|+\lambda_{n}\left\|{ℜ}_{3}\omega_{n}-\eta \right\|\\ & =\left\|\omega_{n}-\eta \right\|. \end{array}$$

This implies that $\left\{\left\|\omega_{n}-\eta \right\|\right\}$ is a non-increasing sequence and is also bounded below for every η∈ℵ. Therefore $\lim_{n\to \infty }\left\|\omega_{n}-\eta \right\|$ exists. □

Now, we apply Lemma 2.2 to establish a necessary and sufficient condition for the existence of a common FP. This condition applies specifically to a set of three *C*-α-NE mappings.

**Theorem 2.3.** *Let* Ω≠∅ *be a bounded, closed, and convex subset of a uniformly convex BS* Ξ*. Suppose that* ${ℜ}_{1},{ℜ}_{2},{ℜ}_{3}:\Omega \to \Omega$ *are three C-*α*-NE mappings, and let* $\left\{\omega_{n}\right\}$ *be the sequence generated by the MZI scheme* (7)*. Then the common FP set*


$$\begin{array}{c} ℵ:=\mathrm{Fix}({ℜ}_{1})\cap \mathrm{Fix}({ℜ}_{2})\cap \mathrm{Fix}({ℜ}_{3}) \end{array}$$

*is nonempty if and only if*


$$\begin{array}{c} \lim_{n\to \infty }\|{ℜ}_{1}\omega_{n}-\omega_{n}\|=0,\;\lim_{n\to \infty }\|{ℜ}_{2}\omega_{n}-\omega_{n}\|=0,\;\text{and}\;\lim_{n\to \infty }\|{ℜ}_{3}\omega_{n}-\omega_{n}\|=0. \end{array}$$

*Proof.* For η∈ℵ, it follows from Lemma 2.2 that, $\lim_{n\to \infty }\left\|\omega_{n}-\eta \right\|$ exists. We can therefore define $\upsilon =\lim_{n\to \infty }\left\|\omega_{n}-\eta \right\|$, where υ is a non-negative real number. Because ${ℜ}_{1},$ ${ℜ}_{2},$ and ${ℜ}_{3}$ are C−α−NE mappings, we get $\underset{n\to \infty }{lim sup}\left\|{ℜ}_{1}\omega_{n}-\xi \right\|\leq \upsilon ,$ $\underset{n\to \infty }{lim sup}\left\|{ℜ}_{2}\omega_{n}-\xi \right\|\leq \upsilon ,$ and $\underset{n\to \infty }{lim sup}\left\|{ℜ}_{3}\omega_{n}-\xi \right\|\leq \upsilon .$ Moreover,


$$\begin{array}{cc} \left\|\kappa_{n}-\eta \right\| & =\left\|{ℜ}_{3}\left[\left(1-\lambda_{n}\right)\omega_{n}+\lambda_{n}{ℜ}_{3}\omega_{n}\right]-\eta \right\|\\ & \leq \left\|\left(1-\lambda_{n}\right)\omega_{n}+\lambda_{n}{ℜ}_{3}\omega_{n}-\eta \right\|\\ & =\left\|\left(1-\lambda_{n}\right)\left(\omega_{n}-\eta \right)+\lambda_{n}\left({ℜ}_{3}\omega_{n}-\eta \right)\right\|\\ & \leq \left(1-\lambda_{n}\right)\left\|\omega_{n}-\eta \right\|+\lambda_{n}\left\|{ℜ}_{3}\omega_{n}-\eta \right\|\\ & \leq \left(1-\lambda_{n}\right)\left\|\omega_{n}-\eta \right\|+\lambda_{n}\left\|\omega_{n}-\eta \right\|\\ & =\left\|\omega_{n}-\eta \right\|, \end{array}$$

and, we have


$$\begin{array}{c} \underset{n\to \infty }{lim sup}\left\|\kappa_{n}-\eta \right\|\leq \underset{n\to \infty }{lim sup}\left\|\omega_{n}-\eta \right\|\leq \upsilon . \end{array}$$ (8)

In addition to, one has


$$\begin{array}{c} \underset{n\to \infty }{lim sup}\left\|{ℜ}_{2}\kappa_{n}-\eta \right\|\leq \underset{n\to \infty }{lim sup}\left\|{ℜ}_{2}\omega_{n}-\eta \right\|\leq \upsilon . \end{array}$$ (9)

Analogously, application of (8) leads to


$$\begin{array}{cc} \underset{n\to \infty }{lim sup}\left\|{ℜ}_{2}\tau_{n}-\eta \right\| & \leq \underset{n\to \infty }{lim sup}\left\|\tau_{n}-\eta \right\|\\ & =\underset{n\to \infty }{lim sup}\left\|\left(1-\xi_{n}\right)\kappa_{n}+\xi_{n}{ℜ}_{2}\kappa_{n}-\eta \right\|\\ & \leq \left(1-\xi_{n}\right)\underset{n\to \infty }{lim sup}\left\|\kappa_{n}-\eta \right\|+\xi_{n}\underset{n\to \infty }{lim sup}\left\|{ℜ}_{2}\kappa_{n}-\eta \right\|\\ & \leq \underset{n\to \infty }{lim sup}\left\|\kappa_{n}-\eta \right\|\\ & \leq \upsilon , \end{array}$$

$$\begin{array}{cc} \underset{n\to \infty }{lim sup}\left\|{ℜ}_{1}\nu_{n}-\eta \right\| & \leq \underset{n\to \infty }{lim sup}\left\|\nu_{n}-\eta \right\|\\ & =\underset{n\to \infty }{lim sup}\left\|\left(1-\rho_{n}\right)\tau_{n}+\rho_{n}{ℜ}_{1}\tau_{n}-\eta \right\|\\ & \leq \left(1-\rho_{n}\right)\underset{n\to \infty }{lim sup}\left\|\tau_{n}-\eta \right\|+\rho_{n}\underset{n\to \infty }{lim sup}\left\|{ℜ}_{1}\tau_{n}-\eta \right\|\\ & \leq \underset{n\to \infty }{lim sup}\left\|\kappa_{n}-\eta \right\|\\ & \leq \upsilon , \end{array}$$

and


$$\begin{array}{cc} \upsilon & =\lim_{n\to \infty }\left\|\omega_{n+1}-\eta \right\|\\ & =\lim_{n\to \infty }\left\|{ℜ}_{3}\nu_{n}-\eta \right\|\\ & \leq \lim_{n\to \infty }\left\|\nu_{n}-\eta \right\|\\ & \leq \lim_{n\to \infty }\left\|\tau_{n}-\eta \right\|\\ & =\lim_{n\to \infty }\left\|\left(1-\xi_{n}\right)\kappa_{n}+\xi_{n}{ℜ}_{2}\kappa_{n}-\eta \right\|\\ & =\lim_{n\to \infty }\left\|\left(1-\xi_{n}\right)\left(\kappa_{n}-\eta \right)+\xi_{n}\left({ℜ}_{2}\kappa_{n}-\eta \right)\right\|\\ & \leq \left(1-\xi_{n}\right)\lim_{n\to \infty }\left\|\left(\kappa_{n}-\eta \right)\right\|+\xi_{n}\lim_{n\to \infty }\left\|\kappa_{n}-\eta \right\|\\ & =\lim_{n\to \infty }\left\|\kappa_{n}-\eta \right\|\\ & \leq \upsilon . \end{array}$$

Hence,


$$\begin{array}{c} \lim_{n\to \infty }\left\|\left(1-\xi_{n}\right)\left(\kappa_{n}-\eta \right)+\xi_{n}\left({ℜ}_{2}\kappa_{n}-\eta \right)\right\|=\upsilon . \end{array}$$ (10)

It follows from (9), (10), and Lemma 1.3 that


$$\begin{array}{c} \lim_{n\to \infty }\left\|\kappa_{n}-{ℜ}_{2}\kappa_{n}\right\|=0. \end{array}$$ (11)

Similarly,


$$\begin{array}{c} \lim_{n\to \infty }\left\|\tau_{n}-{ℜ}_{1}\tau_{n}\right\|=0. \end{array}$$ (12)

Now,


$$\begin{array}{cc} \left\|\omega_{n+1}-\eta \right\| & =\left\|{ℜ}_{3}\nu_{n}-\eta \right\|\\ & \leq \left\|\nu_{n}-\eta \right\|\\ & =\left\|\left(1-\rho_{n}\right)\tau_{n}+\rho_{n}{ℜ}_{1}\tau_{n}-\eta \right\|\\ & \leq \left\|\tau_{n}-\eta \right\|\\ & =\left\|\left(1-\xi_{n}\right)\kappa_{n}+\xi_{n}{ℜ}_{2}\kappa_{n}-\eta \right\|\\ & \leq \left\|\kappa_{n}-\eta \right\|, \end{array}$$

which implies that


$$\begin{array}{c} \upsilon \leq \underset{n\to \infty }{lim sup}\left\|\kappa_{n}-\eta \right\|. \end{array}$$ (13)

Using (9) and (13), we have


$$\begin{array}{c} \lim_{n\to \infty }\left\|\kappa_{n}-\eta \right\|=\upsilon . \end{array}$$

Again


$$\begin{array}{cc} \upsilon & =\lim_{n\to \infty }\left\|\kappa_{n}-\eta \right\|\\ & =\lim_{n\to \infty }\left\|{ℜ}_{3}\left[\left(1-\lambda_{n}\right)\omega_{n}+\lambda_{n}{ℜ}_{3}\omega_{n}\right]-\eta \right\|\\ & \leq \lim_{n\to \infty }\left\|\left(1-\lambda_{n}\right)\left(\omega_{n}-\eta \right)+\lambda_{n}\left({ℜ}_{3}\omega_{n}-\eta \right)\right\|\\ & \leq \left(1-\lambda_{n}\right)\lim_{n\to \infty }\left\|\omega_{n}-\eta \right\|+\lambda_{n}\left\|\omega_{n}-\eta \right\|\\ & \leq \upsilon . \end{array}$$

Hence,


$$\begin{array}{c} \lim_{n\to \infty }\left\|\left(1-\lambda_{n}\right)\left(\omega_{n}-\eta \right)+\lambda_{n}\left({ℜ}_{3}\omega_{n}-\eta \right)\right\|=\upsilon . \end{array}$$ (14)

Applying Lemma 1.3 on (14), one has


$$\begin{array}{c} \lim_{n\to \infty }\left\|\omega_{n}-{ℜ}_{3}\omega_{n}\right\|=0. \end{array}$$ (15)

Hence,


$$\begin{array}{cc} \left\|\omega_{n}-{ℜ}_{3}\kappa_{n}\right\| & \leq \left\|\omega_{n}-{ℜ}_{3}\omega_{n}\right\|+\left\|{ℜ}_{3}\omega_{n}-{ℜ}_{3}\kappa_{n}\right\|\\ & \leq \left\|\omega_{n}-{ℜ}_{3}\omega_{n}\right\|+\left\|\omega_{n}-\kappa_{n}\right\|\\ & =\left\|\omega_{n}-{ℜ}_{3}\omega_{n}\right\|+\left\|{ℜ}_{3}\left[\left(1-\lambda_{n}\right)\omega_{n}+\lambda_{n}{ℜ}_{3}\omega_{n}\right]-\omega_{n}\right\|\\ & \leq \left\|\omega_{n}-{ℜ}_{3}\omega_{n}\right\|+\left\|\left(1-\lambda_{n}\right)\omega_{n}+\lambda_{n}{ℜ}_{3}\omega_{n}-\omega_{n}\right\|\\ & =\left\|\omega_{n}-{ℜ}_{3}\omega_{n}\right\|+\lambda_{n}\left\|{ℜ}_{3}\omega_{n}-\omega_{n}\right\|\\ & =(1+\lambda_{n})\left\|{ℜ}_{3}\omega_{n}-\omega_{n}\right\|\to 0\text{ as }n\to \infty . \end{array}$$ (16)

Additionally


$$\begin{array}{cc} \left\|\kappa_{n}-{ℜ}_{3}\omega_{n}\right\| & =\left\|{ℜ}_{3}\left[\left(1-\lambda_{n}\right)\omega_{n}+\lambda_{n}{ℜ}_{3}\omega_{n}\right]-{ℜ}_{3}\omega_{n}\right\|\\ & \leq \left\|\left(1-\lambda_{n}\right)\omega_{n}+\lambda_{n}{ℜ}_{3}\omega_{n}-\omega_{n}\right\|\\ & \leq \lambda_{n}\left\|{ℜ}_{3}\omega_{n}-\omega_{n}\right\|. \end{array}$$

Using (15), we have


$$\begin{array}{c} \lim_{n\to \infty }\left\|\kappa_{n}-{ℜ}_{3}\omega_{n}\right\|=0. \end{array}$$ (17)

On the other hand,


$$\begin{array}{cc} & \left\|\omega_{n}-{ℜ}_{1}\tau_{n}\right\|\\ & \;=\left\|{ℜ}_{1}\tau_{n}-\omega_{n}\right\|\\ & \;\leq \left\|{ℜ}_{1}\tau_{n}-\tau_{n}\right\|+\left\|\tau_{n}-\omega_{n}\right\|\\ & \;=\left\|{ℜ}_{1}\tau_{n}-\tau_{n}\right\|+\left\|\left(1-\xi_{n}\right)\kappa_{n}+\xi_{n}{ℜ}_{2}\kappa_{n}-\omega_{n}\right\|\\ & \;\leq \left\|{ℜ}_{1}\tau_{n}-\tau_{n}\right\|+\left\|\left(\kappa_{n}-\omega_{n}\right)-\xi_{n}\left(\kappa_{n}-{ℜ}_{2}\kappa_{n}\right)\right\|\\ & \;\leq \left\|{ℜ}_{1}\tau_{n}-\tau_{n}\right\|+\xi_{n}\left\|\kappa_{n}-{ℜ}_{2}\kappa_{n}\right\|+\left\|\kappa_{n}-\omega_{n}\right\|\\ & \;=\left\|{ℜ}_{1}\tau_{n}-\tau_{n}\right\|+\xi_{n}\left\|\kappa_{n}-{ℜ}_{2}\kappa_{n}\right\|+\left\|{ℜ}_{3}\left[\left(1-\lambda_{n}\right)\omega_{n}+\lambda_{n}{ℜ}_{3}\omega_{n}\right]-\omega_{n}\right\|\\ & \;\leq \left\|{ℜ}_{1}\tau_{n}-\tau_{n}\right\|+\xi_{n}\left\|\kappa_{n}-{ℜ}_{2}\kappa_{n}\right\|+\left\|\left(1-\lambda_{n}\right)\omega_{n}+\lambda_{n}{ℜ}_{3}\omega_{n}-\omega_{n}\right\|\\ & \;\leq \left\|{ℜ}_{1}\tau_{n}-\tau_{n}\right\|+\xi_{n}\left\|\kappa_{n}-{ℜ}_{2}\kappa_{n}\right\|+\lambda_{n}\left\|{ℜ}_{3}\omega_{n}-\omega_{n}\right\|. \end{array}$$

Using (11), (12) and (15), we have


$$\begin{array}{c} \lim_{n\to \infty }\left\|\omega_{n}-{ℜ}_{1}\tau_{n}\right\|=0. \end{array}$$ (18)

Also,


$$\begin{array}{cc} \left\|\tau_{n}-{ℜ}_{2}\kappa_{n}\right\| & =\left\|\left(1-\xi_{n}\right)\kappa_{n}-\left(1-\xi_{n}\right){ℜ}_{2}\kappa_{n}\right\|\\ & =\left(1-\xi_{n}\right)\left\|\kappa_{n}-{ℜ}_{2}\kappa_{n}\right\|. \end{array}$$

From (11), we have


$$\begin{array}{c} \lim_{n\to \infty }\left\|\tau_{n}-{ℜ}_{2}\kappa_{n}\right\|=0. \end{array}$$ (19)

Moreover,


$$\begin{array}{cc} \left\|\tau_{n}-\omega_{n}\right\| & =\left\|\tau_{n}-{ℜ}_{1}\tau_{n}+{ℜ}_{1}\tau_{n}-\omega_{n}\right\|\\ & \leq \left\|\tau_{n}-{ℜ}_{1}\tau_{n}\right\|+\left\|{ℜ}_{1}\tau_{n}-\omega_{n}\right\|. \end{array}$$

Applying (12) and (19), we have


$$\begin{array}{c} \lim_{n\to \infty }\left\|\tau_{n}-\omega_{n}\right\|=0. \end{array}$$ (20)

Utilizing Definition 1.1, one can write


$$\begin{array}{cc} {\left\|{ℜ}_{1}\omega_{n}-\omega_{n}\right\|}^{2} & ={\left\|\omega_{n}-{ℜ}_{1}\omega_{n}\right\|}^{2}\\ & \leq {\left[\left\|\omega_{n}-{ℜ}_{1}\tau_{n}\right\|+\left\|{ℜ}_{1}\tau_{n}-{ℜ}_{1}\omega_{n}\right\|\right]}^{2}\\ & ={\left\|\omega_{n}-{ℜ}_{1}\tau_{n}\right\|}^{2}+{\left\|{ℜ}_{1}\tau_{n}-{ℜ}_{1}\omega_{n}\right\|}^{2}+2\left\|\omega_{n}-{ℜ}_{1}\tau_{n}\right\|.\left\|{ℜ}_{1}\tau_{n}-{ℜ}_{1}\omega_{n}\right\|\\ & \leq {\left\|\omega_{n}-{ℜ}_{1}\tau_{n}\right\|}^{2}+\alpha {\left\|{ℜ}_{1}\tau_{n}-\omega_{n}\right\|}^{2}+\alpha {\left\|\tau_{n}-{ℜ}_{1}\omega_{n}\right\|}^{2}+(1-2\alpha ){\left\|\tau_{n}-\omega_{n}\right\|}^{2}\\ & \;+2\left\|\omega_{n}-{ℜ}_{1}\tau_{n}\right\|.\left\|{ℜ}_{1}\tau_{n}-{ℜ}_{1}\omega_{n}\right\|\\ & \leq (1+\alpha ){\left\|\omega_{n}-{ℜ}_{1}\tau_{n}\right\|}^{2}+\alpha \left[{\left\|\tau_{n}-{ℜ}_{2}\kappa_{n}+{ℜ}_{2}\kappa_{n}-{ℜ}_{1}\omega_{n}\right\|}^{2}\right]\\ & \;+(1-2\alpha ){\left\|\tau_{n}-\omega_{n}\right\|}^{2}+2\left\|\omega_{n}-{ℜ}_{1}\tau_{n}\right\|.\left\|{ℜ}_{1}\tau_{n}-{ℜ}_{1}\omega_{n}\right\|\\ & \leq (1+\alpha ){\left\|\omega_{n}-{ℜ}_{1}\tau_{n}\right\|}^{2}+\alpha {\left\|\tau_{n}-{ℜ}_{2}\kappa_{n}\right\|}^{2}+\alpha {\left\|{ℜ}_{2}\kappa_{n}-{ℜ}_{1}\omega_{n}\right\|}^{2}\\ & \;+2\alpha \left\|\tau_{n}-{ℜ}_{2}\kappa_{n}\right\|.\left\|{ℜ}_{2}\kappa_{n}-{ℜ}_{1}\omega_{n}\right\|+(1-2\alpha ){\left\|\tau_{n}-\omega_{n}\right\|}^{2}\\ & \;+2\left\|\omega_{n}-{ℜ}_{1}\tau_{n}\right\|.\left\|{ℜ}_{1}\tau_{n}-{ℜ}_{1}\omega_{n}\right\|\\ & \leq (1+\alpha ){\left\|\omega_{n}-{ℜ}_{1}\tau_{n}\right\|}^{2}+\alpha {\left\|\tau_{n}-{ℜ}_{2}\kappa_{n}\right\|}^{2}+\alpha \left[{\left\|{ℜ}_{2}\kappa_{n}-\tau_{n}+\tau_{n}-{ℜ}_{1}\omega_{n}\right\|}^{2}\right]\\ & \;+2\alpha \left\|\tau_{n}-{ℜ}_{2}\kappa_{n}\right\|.\left\|{ℜ}_{2}\kappa_{n}-{ℜ}_{1}\omega_{n}\right\|+(1-2\alpha ){\left\|\tau_{n}-{ℜ}_{1}\tau_{n}+{ℜ}_{1}\tau_{n}-\omega_{n}\right\|}^{2}\\ & \;+2\left\|\omega_{n}-{ℜ}_{1}\tau_{n}\right\|.\left\|{ℜ}_{1}\tau_{n}-{ℜ}_{1}\omega_{n}\right\|\\ & \leq (1+\alpha ){\left\|\omega_{n}-{ℜ}_{1}\tau_{n}\right\|}^{2}+2\alpha {\left\|\tau_{n}-{ℜ}_{2}\kappa_{n}\right\|}^{2}+\alpha {\left\|\tau_{n}-{ℜ}_{1}\omega_{n}\right\|}^{2}\\ & \;+2\alpha \left\|\tau_{n}-{ℜ}_{2}\kappa_{n}\right\|\left\|\tau_{n}-{ℜ}_{1}\omega_{n}\right\|+2\alpha \left\|\tau_{n}-{ℜ}_{2}\kappa_{n}\right\|.\left\|{ℜ}_{2}\kappa_{n}-{ℜ}_{1}\omega_{n}\right\|\\ & \;+(1-2\alpha )[{\left\|\tau_{n}-{ℜ}_{1}\tau_{n}\right\|}^{2}+{\left\|{ℜ}_{1}\tau_{n}-\omega_{n}\right\|}^{2}+2\left\|\tau_{n}-{ℜ}_{1}\tau_{n}\right\|\left\|{ℜ}_{1}\tau_{n}-\omega_{n}\right\|]\\ & \;+2\left\|\omega_{n}-{ℜ}_{1}\tau_{n}\right\|.\left\|{ℜ}_{1}\tau_{n}-{ℜ}_{1}\omega_{n}\right\|\\ & \leq (1+\alpha ){\left\|\omega_{n}-{ℜ}_{1}\tau_{n}\right\|}^{2}+2\alpha {\left\|\tau_{n}-{ℜ}_{2}\kappa_{n}\right\|}^{2}+\alpha {\left\|\tau_{n}-\omega_{n}+\omega_{n}-{ℜ}_{1}\omega_{n}\right\|}^{2}\\ & \;+2\alpha \left\|\tau_{n}-{ℜ}_{2}\kappa_{n}\right\|\left\|\tau_{n}-{ℜ}_{1}\omega_{n}\right\|+2\alpha \left\|\tau_{n}-{ℜ}_{2}\kappa_{n}\right\|.\left\|{ℜ}_{2}\kappa_{n}-{ℜ}_{1}\omega_{n}\right\|\\ & \;+(1-2\alpha )[{\left\|\tau_{n}-{ℜ}_{1}\tau_{n}\right\|}^{2}+{\left\|{ℜ}_{1}\tau_{n}-\omega_{n}\right\|}^{2}+2\left\|\tau_{n}-{ℜ}_{1}\tau_{n}\right\|\left\|{ℜ}_{1}\tau_{n}-\omega_{n}\right\|]\\ & \;+2\left\|\omega_{n}-{ℜ}_{1}\tau_{n}\right\|.\left\|{ℜ}_{1}\tau_{n}-{ℜ}_{1}\omega_{n}\right\|\\ & \leq (1+\alpha ){\left\|\omega_{n}-{ℜ}_{1}\tau_{n}\right\|}^{2}+2\alpha {\left\|\tau_{n}-{ℜ}_{2}\kappa_{n}\right\|}^{2}+\alpha [{\left\|\tau_{n}-\omega_{n}\right\|}^{2}+{\left\|\omega_{n}-{ℜ}_{1}\omega_{n}\right\|}^{2}\\ & \;+2\left\|\tau_{n}-\omega_{n}\right\|\left\|\omega_{n}-{ℜ}_{1}\omega_{n}\right\|]+2\alpha [\left\|\tau_{n}-{ℜ}_{2}\kappa_{n}\right\|\left\|\tau_{n}-{ℜ}_{1}\omega_{n}\right\|\\ & \;+\left\|\tau_{n}-{ℜ}_{2}\kappa_{n}\right\|.\left\|{ℜ}_{2}\kappa_{n}-{ℜ}_{1}\omega_{n}\right\|]\\ & \;+(1-2\alpha )[{\left\|\tau_{n}-{ℜ}_{1}\tau_{n}\right\|}^{2}+{\left\|{ℜ}_{1}\tau_{n}-\omega_{n}\right\|}^{2}+2\left\|\tau_{n}-{ℜ}_{1}\tau_{n}\right\|\left\|{ℜ}_{1}\tau_{n}-\omega_{n}\right\|]\\ & \;+2\left\|\omega_{n}-{ℜ}_{1}\tau_{n}\right\|.\left\|{ℜ}_{1}\tau_{n}-{ℜ}_{1}\omega_{n}\right\|. \end{array}$$

Hence,


$$\begin{array}{cc} & (1-\alpha ){\left\|{ℜ}_{1}\omega_{n}-\omega_{n}\right\|}^{2}\\ & \;\leq (1+\alpha ){\left\|\omega_{n}-{ℜ}_{1}\tau_{n}\right\|}^{2}+2\alpha {\left\|\tau_{n}-{ℜ}_{2}\kappa_{n}\right\|}^{2}\\ & \;+\alpha [{\left\|\tau_{n}-\omega_{n}\right\|}^{2}+2\left\|\tau_{n}-\omega_{n}\right\|\left\|\omega_{n}-{ℜ}_{1}\omega_{n}\right\|]\\ & \;+2\alpha [\left\|\tau_{n}-{ℜ}_{2}\kappa_{n}\right\|\left\|\tau_{n}-{ℜ}_{1}\omega_{n}\right\|+\left\|\tau_{n}-{ℜ}_{2}\kappa_{n}\right\|.\left\|{ℜ}_{2}\kappa_{n}-{ℜ}_{1}\omega_{n}\right\|]\\ & \;+(1-2\alpha )[{\left\|\tau_{n}-{ℜ}_{1}\tau_{n}\right\|}^{2}+{\left\|{ℜ}_{1}\tau_{n}-\omega_{n}\right\|}^{2}+2\left\|\tau_{n}-{ℜ}_{1}\tau_{n}\right\|\left\|{ℜ}_{1}\tau_{n}-\omega_{n}\right\|]\\ & \;+2\left\|\omega_{n}-{ℜ}_{1}\tau_{n}\right\|.\left\|{ℜ}_{1}\tau_{n}-{ℜ}_{1}\omega_{n}\right\|. \end{array}$$ (21)

Applying (12), (18), (19) and (20) on (21), we conclude that


$$\begin{array}{c} \lim_{n\to \infty }\left\|{ℜ}_{1}\omega_{n}-\omega_{n}\right\|=0. \end{array}$$ (22)

Moreover


$$\begin{array}{cc} \left\|{ℜ}_{2}\omega_{n}-\omega_{n}\right\| & \leq \left\|\omega_{n}-\tau_{n}+\tau_{n}-{ℜ}_{2}\kappa_{n}+{ℜ}_{2}\kappa_{n}-{ℜ}_{2}\omega_{n}\right\|\\ & \leq \left\|\omega_{n}-\tau_{n}\right\|+\left\|\tau_{n}-{ℜ}_{2}\kappa_{n}\right\|+\left\|{ℜ}_{2}\kappa_{n}-{ℜ}_{2}\omega_{n}\right\|\\ & \leq \left\|\omega_{n}-\tau_{n}\right\|+\left\|\tau_{n}-{ℜ}_{2}\kappa_{n}\right\|+\left\|\kappa_{n}-\omega_{n}\right\|\\ & \leq \left\|\omega_{n}-\tau_{n}\right\|+\left\|\tau_{n}-{ℜ}_{2}\kappa_{n}\right\|+\left\|{ℜ}_{3}\left[\left(1-\lambda_{n}\right)\omega_{n}+\lambda_{n}{ℜ}_{3}\omega_{n}\right]-\omega_{n}\right\|\\ & \leq \left\|\omega_{n}-\tau_{n}\right\|+\left\|\tau_{n}-{ℜ}_{2}\kappa_{n}\right\|+\left\|\left(1-\lambda_{n}\right)\omega_{n}+\lambda_{n}{ℜ}_{3}\omega_{n}-\omega_{n}\right\|\\ & \leq \left\|\omega_{n}-\tau_{n}\right\|+\left\|\tau_{n}-{ℜ}_{2}\kappa_{n}\right\|+\lambda_{n}\left\|{ℜ}_{3}\omega_{n}-\omega_{n}\right\|. \end{array}$$

Using (15), (19) and (20), we get


$$\begin{array}{c} \lim_{n\to \infty }\left\|{ℜ}_{2}\omega_{n}-\omega_{n}\right\|=0. \end{array}$$ (23)

It follows from (15), (22) and (23) that


$$\begin{array}{c} \lim_{n\to \infty }\left\|{ℜ}_{1}\omega_{n}-\omega_{n}\right\|=0,\;\lim_{n\to \infty }\left\|{ℜ}_{2}\omega_{n}-\omega_{n}\right\|=0,\text{ and }\lim_{n\to \infty }\left\|{ℜ}_{3}\omega_{n}-\omega_{n}\right\|=0. \end{array}$$ (24)

Conversely, assume that $\left\{\omega_{n}\right\}$ is bounded such that (24) holds. Suppose also that $\eta \in R\left(\Omega ,\{\omega_{n}\}\right).$ Because $\lim_{n\to \infty }\left\|{ℜ}_{1}\omega_{n}-\omega_{n}\right\|=0,$ there exists $n_{0}\in \mathbb{N}$ such that


$$\begin{array}{c} \frac{1}{2}\left\|\omega_{n}-{ℜ}_{1}\omega_{n}\right\|\leq \left\|\omega_{n}-\eta \right\|,\;\forall n\geq n_{0}. \end{array}$$

By the triangle inequality and Definition 1.1, we find that


$$\begin{array}{cc} {\left\|{ℜ}_{1}\eta -\omega_{n}\right\|}^{2} & \leq {\left\|{ℜ}_{1}\eta -{ℜ}_{1}\omega_{n}+{ℜ}_{1}\omega_{n}-\omega_{n}\right\|}^{2}\\ & ={\left\|{ℜ}_{1}\eta -{ℜ}_{1}\omega_{n}\right\|}^{2}+{\left\|{ℜ}_{1}\omega_{n}-\omega_{n}\right\|}^{2}+2\left\|{ℜ}_{1}\eta -{ℜ}_{1}\omega_{n}\right\|\left\|{ℜ}_{1}\omega_{n}-\omega_{n}\right\|\\ & =\alpha {\left\|{ℜ}_{1}\omega_{n}-\eta \right\|}^{2}+\alpha {\left\|\omega_{n}-{ℜ}_{1}\eta \right\|}^{2}+(1-2\alpha ){\left\|\omega_{n}-\eta \right\|}^{2}+{\left\|{ℜ}_{1}\omega_{n}-\omega_{n}\right\|}^{2}\\ & \;+2\left\|{ℜ}_{1}\eta -{ℜ}_{1}\omega_{n}\right\|\left\|{ℜ}_{1}\omega_{n}-\omega_{n}\right\|. \end{array}$$

Hence


$$\begin{array}{cc} (1-\alpha ){\left\|{ℜ}_{1}\eta -\omega_{n}\right\|}^{2} & \leq \alpha {\left\|{ℜ}_{1}\omega_{n}-\eta \right\|}^{2}+(1-2\alpha ){\left\|\omega_{n}-\eta \right\|}^{2}+{\left\|{ℜ}_{1}\omega_{n}-\omega_{n}\right\|}^{2}\\ & \;+2\left\|{ℜ}_{1}\eta -{ℜ}_{1}\omega_{n}\right\|\left\|{ℜ}_{1}\omega_{n}-\omega_{n}\right\|\\ & =\alpha {\left\|{ℜ}_{1}\omega_{n}-\omega_{n}+\omega_{n}-\eta \right\|}^{2}+(1-2\alpha ){\left\|\omega_{n}-\eta \right\|}^{2}+{\left\|{ℜ}_{1}\omega_{n}-\omega_{n}\right\|}^{2}\\ & \;+2\left\|{ℜ}_{1}\eta -{ℜ}_{1}\omega_{n}\right\|\left\|{ℜ}_{1}\omega_{n}-\omega_{n}\right\|\\ & =\alpha [{\left\|\omega_{n}-\eta \right\|}^{2}+{\left\|\omega_{n}-{ℜ}_{1}\omega_{n}\right\|}^{2}+2\left\|\omega_{n}-\eta \right\|\left\|\omega_{n}-{ℜ}_{1}\omega_{n}\right\|]\\ & \;+(1-2\alpha ){\left\|\omega_{n}-\eta \right\|}^{2}+{\left\|{ℜ}_{1}\omega_{n}-\omega_{n}\right\|}^{2}+2\left\|{ℜ}_{1}\eta -{ℜ}_{1}\omega_{n}\right\|\left\|{ℜ}_{1}\omega_{n}-\omega_{n}\right\|. \end{array}$$

Therefore


$$\begin{array}{cc} {\left\|{ℜ}_{1}\eta -\omega_{n}\right\|}^{2} & \leq \frac{\left(1-\alpha \right)}{\left(1-\alpha \right)}{\left\|\omega_{n}-\eta \right\|}^{2}+\frac{\left(1+\alpha \right)}{\left(1-\alpha \right)}{\left\|\omega_{n}-{ℜ}_{1}\omega_{n}\right\|}^{2}\\ & \;+\frac{2\alpha }{\left(1-\alpha \right)}\left\|\omega_{n}-\eta \right\|\left\|\omega_{n}-{ℜ}_{1}\omega_{n}\right\|\\ & \;+\frac{2}{\left(1-\alpha \right)}\left\|{ℜ}_{1}\eta -{ℜ}_{1}\omega_{n}\right\|\left\|{ℜ}_{1}\omega_{n}-\omega_{n}\right\|. \end{array}$$ (25)

Taking the limsup of both sides of inequality (25) yields


$$\begin{array}{c} \underset{n\to \infty }{lim sup}{\left\|{ℜ}_{1}\eta -\omega_{n}\right\|}^{2}\leq \underset{n\to \infty }{lim sup}{\left\|\eta -\omega_{n}\right\|}^{2}, \end{array}$$

implies


$$\begin{array}{c} \underset{n\to \infty }{lim sup}\left\|{ℜ}_{1}\eta -\omega_{n}\right\|\leq \underset{n\to \infty }{lim sup}\left\|\eta -\omega_{n}\right\|. \end{array}$$ (26)

Utilizing inequality (25) and Definitions 1.4 and 1.5, we deduce that


$$\begin{array}{cc} s\left({ℜ}_{1}\eta ,\{\omega_{n}\}\right) & =\underset{n\to \infty }{lim sup}\left\|{ℜ}_{1}\eta -\omega_{n}\right\|\\ & \leq \underset{n\to \infty }{lim sup}\left\|\eta -\omega_{n}\right\|\\ & =s\left(\eta ,\{\omega_{n}\}\right)\\ & =s\left(\Omega ,\{\omega_{n}\}\right). \end{array}$$

Hence, ${ℜ}_{1}\eta \in R\left(\Omega ,\{\omega_{n}\}\right).$ The uniform convexity of Ξ ensures that $R\left(\Omega ,\{\omega_{n}\}\right)$ is a singleton set. Hence, we obtain ${ℜ}_{1}\eta =\eta$. A similar argument leads to ${ℜ}_{2}\eta =\eta$ and ${ℜ}_{3}\eta =\eta$. Therefore, η∈ℵ, thereby providing additional confirmation that ℵ≠∅. □

We now turn to establishing the weak convergence of the MZI scheme (7) toward a common FP of the mappings ${ℜ}_{1}$, ${ℜ}_{2}$, and ${ℜ}_{3}$.

**Theorem 2.4.** *Assume that* Ξ *is a uniformly convex BS endowed with the Opial property. Let* Ω*,* ${ℜ}_{1}$*,* ${ℜ}_{2}$*, and* ${ℜ}_{3}$ *be as defined in Theorem 2.3. Then the sequence* $\left\{\omega_{n}\right\}$ *generated by the MZI scheme* (7) *converges weakly to a common FP of* ${ℜ}_{1}$*,* ${ℜ}_{2}$*, and* ${ℜ}_{3}$*, provided that*


$$\begin{array}{c} ℵ:=\mathrm{Fix}({ℜ}_{1})\cap \mathrm{Fix}({ℜ}_{2})\cap \mathrm{Fix}({ℜ}_{3}) \end{array}$$

*is nonempty.*

*Proof.* It follows from [17, Theorem 4.1] that, in a uniformly convex BS Ξ with the Opial property, the operators $I-{ℜ}_{1}$, $I-{ℜ}_{2}$, and $I-{ℜ}_{3}$ are demiclosed at zero, provided that ${ℜ}_{1},{ℜ}_{2},{ℜ}_{3}:\Omega \to \Omega$ are *C*-α-NE mappings. The remainder of the proof proceeds along the same lines as in [31, Theorem 3.4]. □

It is worth noting that the Opial property does not hold in many uniformly convex BSs. Therefore, in the subsequent analysis, we adopt an alternative approach by utilizing the existence of a Fréchet differentiable norm and develop a different line of reasoning to replace reliance on the Opial property.

**Theorem 2.5.** *In Theorem 2.4, replace the assumption that* Ξ *has the Opial property with the condition that* Ξ *admits a Fréchet differentiable norm. Additionally, assume that the limit*


$$\begin{array}{c} \lim_{n\to \infty }\|s\omega_{n}+(1-s)\varrho -\varkappa \| \end{array}$$

*exists for all* ϱ,ϰ∈ℵ *and for some fixed* s∈(0,1)*, and that the operators* $I-{ℜ}_{1}$, $I-{ℜ}_{2}$*, and* $I-{ℜ}_{3}$ *are demiclosed at zero. Then the sequence* $\left\{\omega_{n}\right\}$ *generated by the MZI scheme (7) converges weakly to a common FP of* ${ℜ}_{1}$, ${ℜ}_{2}$*, and* ${ℜ}_{3}$.

*Proof.* The result follows by applying the argument used in the proof of [31, Theorem 3.5]. □

We now generalize the results of Bera et al. [31] and Chidume and Ali [34] by introducing the following condition, denoted as $\left(B^{\prime }\right)$.

We say that the mappings ${ℜ}_{1},{ℜ}_{2},{ℜ}_{3}:\Omega \to \Omega$ satisfy *Condition* $\left(B^{\prime }\right)$ with ℵ≠∅ if there exists a nondecreasing function h:[0,∞)→[0,∞) such that *h*(0) = 0 and h(ϑ)>0 for all ϑ∈(0,∞), and for all ω∈Ω, the following inequality holds:


$$\begin{array}{c} \max\left\{\|\omega -{ℜ}_{1}\omega \|,\|\omega -{ℜ}_{2}\omega \|,\|\omega -{ℜ}_{3}\omega \|\right\}\geq h\left(d(\omega ,ℵ)\right), \end{array}$$

where


$$\begin{array}{c} d(\omega ,ℵ):=\inf_{\eta \in ℵ}\|\omega -\eta \|. \end{array}$$

**Lemma 4 ([31]).** *Let* $\left\{\gamma_{n}\right\}$ *and* $\left\{\varpi_{n}\right\}$ *be sequences of nonnegative real numbers such that*


$$\begin{array}{c} \gamma_{n+1}\leq \gamma_{n}+\varpi_{n}\;\text{for all }n\in \mathbb{N}, \end{array}$$

*and suppose that* $\sum_{n=0}^{\infty }\varpi_{n}<\infty$*. Then the limit* $\lim_{n\to \infty }\gamma_{n}$ *exists.*

Using the above lemma, we now establish a strong convergence result for the MZI scheme.

**Theorem 2.7.** *Let* Ω≠∅ *be a bounded, closed, and convex subset of a uniformly convex BS* Ξ*. Suppose that* ${ℜ}_{1},{ℜ}_{2},{ℜ}_{3}:\Omega \to \Omega$ *are three C-*α*-NE mappings with*


$$\begin{array}{c} ℵ:=\mathrm{Fix}({ℜ}_{1})\cap \mathrm{Fix}({ℜ}_{2})\cap \mathrm{Fix}({ℜ}_{3})\neq \emptyset , \end{array}$$

*and let* $\left\{\omega_{n}\right\}$ *be the sequence generated by the MZI scheme (7). Then* $\left\{\omega_{n}\right\}$ *converges strongly to a common FP of* ${ℜ}_{1}$, ${ℜ}_{2}$*, and* ${ℜ}_{3}$*, provided that the mappings satisfy Condition* $\left(B^{\prime }\right)$.

*Proof.* Assume that η∈ℵ. Thanks to Lemma 2.2, $\lim_{n\to \infty }\left\|\omega_{n}-\eta \right\|$ exists. Also, $\left\|\omega_{n+1}-\eta \right\|\leq \left\|\omega_{n}-\eta \right\|$ for all n=0,1,2,⋯.

It follows that


$$\begin{array}{c} d\left(\omega_{n+1},ℵ\right)\leq d\left(\omega_{n},ℵ\right). \end{array}$$

Consequently, Lemma 2.6 guarantees the existence of $\lim_{n\to \infty }d\left(\omega_{n},ℵ\right)$ In addition, we know from Theorem 2.3 that (24) holds.

The adherence of ${ℜ}_{1},$ ${ℜ}_{2},$ and ${ℜ}_{3}$ to Condition $\left(B^{\prime }\right)$ directly implies that


$$\begin{array}{c} \lim_{n\to \infty }h\left(d\left(\omega_{n},ℵ\right)\right)=0, \end{array}$$

for a nondecreasing function *h*. This result then leads to


$$\begin{array}{c} \lim_{n\to \infty }d\left(\omega_{n},ℵ\right)=0. \end{array}$$

Consequently, we can select a subsequence $\left\{\omega_{n_{i}}\right\}$ of $\left\{\omega_{n}\right\}$ and a corresponding sequence $\left\{\eta_{i}\right\}$ in ℵ such that $\left\|\omega_{n_{i}}-\eta_{i}\right\|<\frac{\varepsilon }{2}$ for all i∈ℕ and any given ε>0. To show that $\left\{\omega_{n}\right\}$ is Cauchy, we consider n,ϑ≥i. In this case, we get


$$\begin{array}{cc} \left\|\omega_{n+\vartheta }-\omega_{n}\right\| & \leq \left\|\omega_{n+\vartheta }-\eta_{i}\right\|+\left\|\eta_{i}-\omega_{n}\right\|\\ & \leq \left\|\omega_{n+\vartheta -1}-\eta_{i}\right\|+\left\|\eta_{i}-\omega_{n}\right\|\\ & \leq \left\|\omega_{n+\vartheta -2}-\eta_{i}\right\|+\left\|\eta_{i}-\omega_{n}\right\|\\ & \;\vdots \\ & \leq 2\left\|\eta_{i}-\omega_{n}\right\|\\ & =2\left\|\omega_{n_{i}}-\omega_{n}\right\|<\epsilon . \end{array}$$

Therefore $\left\{\omega_{n}\right\}$ is Cauchy sequence in Ω. Since Ω is closed in Ξ, we know that $\lim_{n\to \infty }\omega_{n}=\delta$ for any δ∈Ω. The fact that ℵ is closed and $\lim_{n\to \infty }d\left(\omega_{n},ℵ\right)=0$ then leads directly to the conclusion that δ∈ℵ. This completes the proof. □

**Remark 1.** *Since the class of C-*α*-NE mappings properly contains the class of* α*-NE mappings, the results presented in this work constitute a meaningful extension of the existing literature. In particular, our findings refine, complement, and generalize the results of Muangchoo-in et al.* [35] *and Naraghirad* [11] *in Banach spaces, while also extending the work of Pant and Shukla* [19]*.*

## 3 Practical comparisons

This section presents a comparative analysis of convergence rates. Specifically, we compare the performance of the proposed *Z*-iterative scheme (7) with several established iterative methods, namely those of Noor [36], S [37], Ishikawa [18], Abbas–Nazir [20], and HR [38]. The setting is a BS Ξ, where ${ℜ}_{1}$ and ${ℜ}_{2}$ are contraction mappings (CAMs) defined on a nonempty, closed, and convex subset Ω⊂Ξ. We assume that the sequences $\left\{\rho_{n}\right\}$, $\left\{\xi_{n}\right\}$, and $\left\{\lambda_{n}\right\}$ are chosen from the interval (0,1). Starting from arbitrary initial points $\varpi_{0},s_{0},\vartheta_{0},g_{0},\varrho_{0}\in \Omega$, the iterative schemes are defined as follows:

Noor iteration [36]:


$$\begin{array}{c} \left\{ \begin{array}{cc} & \varpi_{0}\in \Omega ,\\ & \kappa_{n}=(1-\lambda_{n})\varpi_{n}+\lambda_{n}{ℜ}_{1}\omega_{n},\\ & \tau_{n}=(1-\xi_{n})\varpi_{n}+\xi_{n}{ℜ}_{2}\kappa_{n},\\ & \varpi_{n+1}=(1-\rho_{n})\varpi_{n}+\rho_{n}{ℜ}_{1}\tau_{n}. \end{array}\right. \end{array}$$ (27)

S-iteration [37]:


$$\begin{array}{c} \left\{ \begin{array}{cc} & s_{0}\in \Omega ,\\ & \kappa_{n}=(1-\lambda_{n})s_{n}+\lambda_{n}{ℜ}_{1}s_{n},\\ & \tau_{n}=(1-\xi_{n})\kappa_{n}+\xi_{n}{ℜ}_{2}\kappa_{n},\\ & s_{n+1}=(1-\rho_{n}){ℜ}_{1}s_{n}+\rho_{n}{ℜ}_{2}\tau_{n}. \end{array}\right. \end{array}$$ (28)

Ishikawa iteration [18]:


$$\begin{array}{c} \left\{ \begin{array}{cc} & \vartheta_{0}\in \Omega ,\\ & \tau_{n}=(1-\xi_{n})\vartheta_{n}+\xi_{n}{ℜ}_{2}\vartheta_{n},\\ & \vartheta_{n+1}=(1-\rho_{n})\vartheta_{n}+\rho_{n}{ℜ}_{1}\tau_{n}. \end{array}\right. \end{array}$$ (29)

Abbas-Nazir iteration [20]:


$$\begin{array}{c} \left\{ \begin{array}{cc} & g_{0}\in \Omega ,\\ & \kappa_{n}=(1-\lambda_{n})g_{n}+\lambda_{n}{ℜ}_{1}g_{n},\\ & \tau_{n}=(1-\xi_{n})g_{n}+\xi_{n}{ℜ}_{2}\kappa_{n},\\ & g_{n+1}=(1-\rho_{n}){ℜ}_{1}\tau_{n}+\rho_{n}{ℜ}_{2}\kappa_{n}. \end{array}\right. \end{array}$$ (30)

HR-iteration [38]:


$$\begin{array}{c} \left\{ \begin{array}{cc} & \varrho_{0}\in \Omega ,\\ & \omega_{n}=(1-\rho_{n})\varrho_{n}+\rho_{n}{ℜ}_{1}\varrho_{n},\\ & \tau_{n}={ℜ}_{2}\left[(1-\xi_{n})\omega_{n}+\xi_{n}{ℜ}_{2}\omega_{n}\right],\\ & \kappa_{n}={ℜ}_{2}\left[(1-\lambda_{n})\tau_{n}+\lambda_{n}{ℜ}_{2}\tau_{n}\right],\\ & \varrho_{n+1}={ℜ}_{2}\kappa_{n}, \end{array}\right. \end{array}$$ (31)

for all n=0,1,2,….

In the following, we analyze the convergence behavior of the MZI scheme (7) when applied to three contraction mappings (CAMs).

**Theorem 3.1.** *Let* Ω≠∅ *be a closed convex subset of a Banach space* Ξ*. Suppose that* ${ℜ}_{1},{ℜ}_{2},{ℜ}_{3}:\Omega \to \Omega$ *are three CAMs with a nonempty common FP set*


$$\begin{array}{c} ℵ:=\mathrm{Fix}({ℜ}_{1})\cap \mathrm{Fix}({ℜ}_{2})\cap \mathrm{Fix}({ℜ}_{3})\neq \emptyset , \end{array}$$

*and let* $\left\{\omega_{n}\right\}$ *be the sequence generated by the MZI scheme (7), where the control sequences* $\left\{\rho_{n}\right\}$, $\left\{\xi_{n}\right\}$*, and* $\left\{\lambda_{n}\right\}$ *are in (0,1). Let* η∈ℵ *be a common FP of* ${ℜ}_{1}$, ${ℜ}_{2}$*, and* ${ℜ}_{3}$*. Then* $\left\{\omega_{n}\right\}$ *converges strongly to the unique common FP of* ${ℜ}_{1}$, ${ℜ}_{2}$*, and* ${ℜ}_{3}$ *provided that the following conditions are satisfied:*

(i) $\sum_{n=0}^{\infty }\rho_{n}=\infty \backslash )$, $\sum_{n=0}^{\infty }\xi_{n}=\infty \backslash )$, $\sum_{n=0}^{\infty }\lambda_{n}=\infty \backslash )$,

(ii) $\sum_{n=0}^{\infty }\lambda_{n}\xi_{n}\rho_{n}=\infty \backslash )$.

*Proof.* From the MZI (7), we get


$$\begin{array}{cc} \left\|\nu_{n}-\eta \right\| & =\left\|\left(1-\rho_{n}\right)\tau_{n}+\rho_{n}{ℜ}_{1}\tau_{n}-\eta \right\|\\ & \leq \left(1-\rho_{n}\right)\left\|\tau_{n}-\eta \right\|+\rho_{n}\left\|{ℜ}_{1}\tau_{n}-{ℜ}_{1}\eta \right\|\\ & \leq \left(1-\rho_{n}\right)\left\|\tau_{n}-\eta \right\|+\rho_{n}\varsigma \left\|\tau_{n}-\eta \right\|\\ & =\left(1-\rho_{n}+\rho_{n}\varsigma \right)\left\|\tau_{n}-\eta \right\|\\ & =\left[1-\rho_{n}(1-\varsigma )\right]\left\|\tau_{n}-\eta \right\|, \end{array}$$ (32)

$$\begin{array}{cc} \left\|\tau_{n}-\eta \right\| & =\left\|\left(1-\xi_{n}\right)\kappa_{n}+\xi_{n}{ℜ}_{2}\kappa_{n}-\eta \right\|\\ & \leq \left(1-\xi_{n}\right)\left\|\kappa_{n}-\eta \right\|+\xi_{n}\left\|{ℜ}_{2}\kappa_{n}-{ℜ}_{2}\eta \right\|\\ & \leq \left[1-\xi_{n}(1-\varsigma )\right]\left\|\kappa_{n}-\eta \right\|, \end{array}$$ (33)

and


$$\begin{array}{cc} \left\|\kappa_{n}-\eta \right\| & =\left\|{ℜ}_{3}\left[\left(1-\lambda_{n}\right)\omega_{n}+\lambda_{n}{ℜ}_{3}\omega_{n}\right]-{ℜ}_{3}\eta \right\|\\ & \leq \varsigma \left\|\left(1-\lambda_{n}\right)\omega_{n}+\lambda_{n}{ℜ}_{3}\omega_{n}-\eta \right\|\\ & \leq \varsigma \left(1-\lambda_{n}\right)\left\|\omega_{n}-\eta \right\|+\varsigma \lambda_{n}\left\|{ℜ}_{3}\omega_{n}-{ℜ}_{3}\eta \right\|\\ & \leq \varsigma \left(1-\lambda_{n}\right)\left\|\omega_{n}-\eta \right\|+\varsigma^{2}\lambda_{n}\left\|\omega_{n}-\eta \right\|\\ & =\left[\varsigma -\varsigma \lambda_{n}+\varsigma^{2}\lambda_{n}\right]\left\|\omega_{n}-\eta \right\|\\ & =\varsigma \left[1-\lambda_{n}(1-\varsigma )\right]\left\|\omega_{n}-\eta \right\|. \end{array}$$ (34)

It follows from (32), (33) and (34) that


$$\begin{array}{cc} \left\|\omega_{n+1}-\eta \right\| & =\left\|{ℜ}_{3}\nu_{n}-\eta \right\|\\ & =\left\|{ℜ}_{3}\nu_{n}-{ℜ}_{3}\eta \right\|\\ & \leq \varsigma \left\|\nu_{n}-\eta \right\|\\ & \leq \varsigma \left[1-\rho_{n}(1-\varsigma )\right]\left\|\tau_{n}-\eta \right\|\\ & \leq \varsigma \left[1-\rho_{n}(1-\varsigma )\right]\left[1-\xi_{n}(1-\varsigma )\right]\left\|\kappa_{n}-\eta \right\|\\ & \leq \varsigma \left[1-\rho_{n}(1-\varsigma )\right]\left[1-\xi_{n}(1-\varsigma )\right]\left[1-\lambda_{n}(1-\varsigma )\right]\left\|\omega_{n}-\eta \right\|. \end{array}$$

Therefore, for ς∈(0,1), we have


$$\begin{array}{cc} & \left\|\omega_{n+1}-\eta \right\|\\ & \;\leq \varsigma^{2}\left[1-\left[\rho_{n}+\xi_{n}+\lambda_{n}-\left(\xi_{n}\lambda_{n}+\left[\rho_{n}\lambda_{n}+\rho_{n}\xi_{n}\right](1-\varsigma )\right)-\rho_{n}\xi_{n}\lambda_{n}(1-\varsigma {)}^{2}\right](1-\varsigma )\right]\left\|\omega_{n}-\eta \right\|. \end{array}$$

Following these steps iteratively, we find that


$$\begin{array}{cc} \left\|\omega_{n}-\eta \right\| & \leq \varsigma^{2}[1-\{\rho_{n-1}+\xi_{n-1}+\lambda_{n-1}-\left(\xi_{n-1}\lambda_{n-1}+\left[\rho_{n-1}\lambda_{n-1}+\rho_{n-1}\xi_{n-1}\right](1-\varsigma )\right)\\ & \;-\rho_{n-1}\xi_{n-1}\lambda_{n-1}(1-\varsigma {)}^{2}\}(1-\varsigma )]\left\|\omega_{n-1}-\eta \right\|, \end{array}$$

which implies that


$$\begin{array}{cc} \left\|\omega_{n-1}-\eta \right\| & \leq \varsigma^{2}[1-\{\rho_{n-2}+\xi_{n-2}+\lambda_{n-2}-\left(\xi_{n-2}\lambda_{n-2}+\left[\rho_{n-2}\lambda_{n-2}+\rho_{n-2}\xi_{n-2}\right](1-\varsigma )\right)\\ & \;-\rho_{n-2}\xi_{n-2}\lambda_{n-2}(1-\varsigma {)}^{2}\}(1-\varsigma )]\left\|\omega_{n-2}-\eta \right\|,\\ & \;\vdots \\ \left\|\omega_{1}-\eta \right\| & \leq \varsigma^{2}[1-\{\rho_{0}+\xi_{0}+\lambda_{0}-\left(\xi_{0}\lambda_{0}+\left[\rho_{0}\lambda_{0}+\rho_{0}\xi_{0}\right](1-\varsigma )\right)\\ & \;-\rho_{0}\xi_{0}\lambda_{0}(1-\varsigma {)}^{2}\}(1-\varsigma )]\left\|\omega_{0}-\eta \right\|, \end{array}$$

it follows inductively that


$$\begin{array}{cc} \left\|\omega_{n+1}-\eta \right\| & \leq \varsigma^{2(n+1)}\prod_{j=0}^{n}[1-\{\rho_{j}+\xi_{j}+\lambda_{j}-\left(\xi_{j}\lambda_{j}+\left[\rho_{j}\lambda_{j}+\rho_{j}\xi_{j}\right](1-\varsigma )\right)\\ & \;-\rho_{j}\xi_{j}\lambda_{j}(1-\varsigma {)}^{2}\}(1-\varsigma )]\left\|\omega_{0}-\eta \right\|. \end{array}$$ (35)

Using the fact that $1-\omega \leq e^{-\omega },$ we have


$$\begin{array}{c} \left\|\omega_{n+1}-\eta \right\|\leq \varsigma^{2}e^{-(1-\varsigma )\sum_{j=0}^{\infty }\left(\rho_{j}+\xi_{j}+\lambda_{j}-\left(\xi_{j}\lambda_{j}+\left[\rho_{j}\lambda_{j}+\rho_{j}\xi_{j}\right](1-\varsigma )\right)-\rho_{j}\xi_{j}\lambda_{j}(1-\varsigma {)}^{2}\right)}\left\|\omega_{0}-\eta \right\|. \end{array}$$

Applying the assumptions i), and ii) of Theorem 3.1 and passing n→∞, we conclude that


$$\begin{array}{c} \lim_{n\to \infty }\left\|\omega_{n}-\eta \right\|=0. \end{array}$$

Since $\varsigma^{2}<\varsigma <1,$ the sequence $\left\{\omega_{n}\right\}$ converges strongly to η. □

We now provide an analytical comparison of convergence rates for CAMs, thereby highlighting the superior performance of the proposed MZI scheme (7) relative to several well-established iterative methods.

**Theorem 3.2.** *Let* Ω≠∅ *be a closed convex subset of a BS* Ξ*, and let* ${ℜ}_{1},{ℜ}_{2},{ℜ}_{3}:\Omega \to \Omega$ *be CAMs such that the common FP set*


$$\begin{array}{c} ℵ:=\mathrm{Fix}({ℜ}_{1})\cap \mathrm{Fix}({ℜ}_{2})\cap \mathrm{Fix}({ℜ}_{3}) \end{array}$$

*is nonempty. Let* η∈ℵ *be a common FP of* ${ℜ}_{1}$, ${ℜ}_{2}$*, and* ${ℜ}_{3}$*. Consider the sequences* $\left\{\omega_{n}\right\}$, $\left\{\varpi_{n}\right\}$, $\left\{s_{n}\right\}$, $\left\{\vartheta_{n}\right\}$, $\left\{g_{n}\right\}$*, and* $\left\{\varrho_{n}\right\}$ *generated by the MZI scheme (7), and the Noor (27), S (28), Ishikawa (29), Abbas–Nazir (30), and HR (31) schemes, respectively. Suppose the control sequences* $\left\{\rho_{n}\right\}$, $\left\{\xi_{n}\right\}$*, and* $\left\{\lambda_{n}\right\}$ *lie in (0,1) and satisfy*


$$\begin{array}{c} \lim_{n\to \infty }\rho_{n}=0,\;\lim_{n\to \infty }\xi_{n}=0,\;\text{and}\;\lim_{n\to \infty }\lambda_{n}=0. \end{array}$$

*Then, the MZI scheme (7) converges to* η *faster than each of the Noor (27), S (28), Ishikawa (29), Abbas–Nazir (30), and HR (31) iterative schemes.*

*Proof.* Applying the same calculation method as in Theorem 2.3, we derive the following estimates for the Noor iterative scheme (27):


$$\begin{array}{cc} \left\|\kappa_{n}-\eta \right\| & =\left\|\left(1-\lambda_{n}\right)\varpi_{n}+\lambda_{n}{ℜ}_{1}\omega_{n}-\eta \right\|\\ & \leq \left(1-\lambda_{n}\right)\left\|\varpi_{n}-\eta \right\|+\lambda_{n}\left\|{ℜ}_{1}\omega_{n}-\eta \right\|\\ & \leq \left(1-\lambda_{n}\right)\left\|\varpi_{n}-\eta \right\|+\lambda_{n}\varsigma \left\|\omega_{n}-\eta \right\|\\ & =\left(1-\lambda_{n}(1-\varsigma )\right)\left\|\omega_{n}-\eta \right\|, \end{array}$$

$$\begin{array}{cc} \left\|\tau_{n}-\eta \right\| & =\left\|\left(1-\xi_{n}\right)\varpi_{n}+\xi_{n}{ℜ}_{2}\kappa_{n}-\eta \right\|\\ & \leq \left(1-\xi_{n}\right)\left\|\varpi_{n}-\eta \right\|+\xi_{n}\left\|{ℜ}_{1}\kappa_{n}-\eta \right\|\\ & \leq \left(1-\xi_{n}\right)\left\|\varpi_{n}-\eta \right\|+\xi_{n}\varsigma \left\|\kappa_{n}-\eta \right\|\\ & \leq \left(1-\xi_{n}\right)\left\|\varpi_{n}-\eta \right\|+\xi_{n}\varsigma \left(1-\lambda_{n}(1-\varsigma )\right)\left\|\omega_{n}-\eta \right\|\\ & \leq \left[1-\xi_{n}(1-\varsigma (1-\lambda_{n}(1-\varsigma )))\right]\left\|\omega_{n}-\eta \right\|, \end{array}$$

and


$$\begin{array}{cc} \left\|\varpi_{n+1}-\eta \right\| & =\left\|\left(1-\rho_{n}\right)\varpi_{n}+\rho_{n}{ℜ}_{1}\tau_{n}-\eta \right\|\\ & =\left\|\left(1-\rho_{n}\right)\left(\varpi_{n}-\eta \right)+\rho_{n}\left({ℜ}_{1}\tau_{n}-\eta \right)\right\|\\ & \geq \left\|\left(1-\rho_{n}\right)\left(\varpi_{n}-\eta \right)-\rho_{n}\left({ℜ}_{1}\tau_{n}-\eta \right)\right\|\\ & \geq \left|\left(1-\rho_{n}\right)\left\|\varpi_{n}-\eta \right\|-\rho_{n}\varsigma \left\|\tau_{n}-\eta \right\|\right|\\ & \geq |\left(1-\rho_{n}\right)\left\|\varpi_{n}-\eta \right\|\\ & \;-\rho_{n}\varsigma \left[1-\xi_{n}(1-\varsigma (1-\lambda_{n}(1-\varsigma )))\right]\left\|\omega_{n}-\eta \right\||\\ & =\left|1-\rho_{n}(1+\varsigma (1-\xi_{n}(1-\varsigma (1-\lambda_{n}(1-\varsigma )))))\left\|\omega_{n}-\eta \right\|\right|\\ & \geq \left|1-\rho_{n}(1+\varsigma )\right|\left\|\omega_{n}-\eta \right\|\\ & =\prod_{j=0}^{n}\left(1-\rho_{j}(1+\varsigma )\right)\left\|\omega_{0}-\eta \right\|. \end{array}$$

For the Noor iterative scheme, we therefore obtain the following estimate:


$$\begin{array}{c} \left\|\varpi_{n+1}-\eta \right\|\geq \prod_{j=0}^{n}\left(1-\rho_{j}(1+\varsigma )\right)\left\|\varpi_{0}-\eta \right\|. \end{array}$$ (36)

Similar to the calculations performed for the MZI (7) in (35) and the Noor iteration (27) in (36), estimates for the S (28), Ishikawa (29), Abbas–Nazir (30), and HR (31) iterative schemes can be determined. These are detailed below.


$$\begin{array}{c} \left\|s_{n+1}-\eta \right\|\geq \varsigma^{\left(1+n\right)}\prod_{j=0}^{n}[1-\rho_{j}(1+\rho_{j}(1-\xi_{j}(1-\varsigma ))(1-\lambda_{j}(1-\varsigma )))]\left\|s_{0}-\eta \right\|, \end{array}$$ (37)

$$\begin{array}{c} \left\|\vartheta_{n+1}-\eta \right\|\geq \prod_{j=0}^{n}[1-\rho_{j}(1+\varsigma )]\left\|\vartheta_{0}-\eta \right\|, \end{array}$$ (38)

$$\begin{array}{c} \left\|g_{n+1}-\eta \right\|\leq \varsigma^{\left(1+n\right)}\prod_{j=0}^{n}[1-\rho_{j}\xi_{j}\lambda_{j}(1-\varsigma )]\left\|g_{0}-\eta \right\|, \end{array}$$ (39)

and


$$\begin{array}{c} \left\|\varrho_{n+1}-\eta \right\|\leq \varsigma^{\left(1+n\right)}\prod_{j=0}^{n}[1-\lambda_{j}(1-\varsigma )]\left\|\varrho_{0}-\eta \right\|. \end{array}$$ (40)

Now, utilizing (35) with (36)-(40), respectively and selecting $\omega_{0}=\varpi_{0}=s_{0}=\vartheta_{0},$ one has


$$\begin{array}{cc} 0 & \leq \frac{\left\|\omega_{n+1}-\eta \right\|}{\left\|\varpi_{n+1}-\eta \right\|}\\ & \leq \frac{\varsigma^{2(n+1)}\prod_{j=0}^{n}\left[1-\left\{\rho_{j}+\xi_{j}+\lambda_{j}-\left(\xi_{j}\lambda_{j}+\left[\rho_{j}\lambda_{j}+\rho_{j}\xi_{j}\right](1-\varsigma )\right)-\rho_{j}\xi_{j}\lambda_{j}(1-\varsigma {)}^{2}\right\}(1-\varsigma )\right]}{\prod_{j=0}^{n}\left(1-\rho_{j}(1+\varsigma )\right)}, \end{array}$$

$$\begin{array}{cc} 0 & \leq \frac{\left\|\omega_{n+1}-\eta \right\|}{\left\|s_{n+1}-\eta \right\|}\\ & \leq \frac{\varsigma^{2(n+1)}\prod_{j=0}^{n}\left[1-\left\{\rho_{j}+\xi_{j}+\lambda_{j}-\left(\xi_{j}\lambda_{j}+\left[\rho_{j}\lambda_{j}+\rho_{j}\xi_{j}\right](1-\varsigma )\right)-\rho_{j}\xi_{j}\lambda_{j}(1-\varsigma {)}^{2}\right\}(1-\varsigma )\right]}{\varsigma^{\left(1+n\right)}\prod_{j=0}^{n}[1-\rho_{j}(1+\rho_{j}(1-\xi_{j}(1-\varsigma ))(1-\lambda_{j}(1-\varsigma )))]}, \end{array}$$

and


$$\begin{array}{cc} 0 & \leq \frac{\left\|\omega_{n+1}-\eta \right\|}{\left\|\vartheta_{n+1}-\eta \right\|}\\ & \leq \frac{\varsigma^{2(n+1)}\prod_{j=0}^{n}\left[1-\left\{\rho_{j}+\xi_{j}+\lambda_{j}-\left(\xi_{j}\lambda_{j}+\left[\rho_{j}\lambda_{j}+\rho_{j}\xi_{j}\right](1-\varsigma )\right)-\rho_{j}\xi_{j}\lambda_{j}(1-\varsigma {)}^{2}\right\}(1-\varsigma )\right]}{\prod_{j=0}^{n}[1-\rho_{j}(1+\varsigma )]}. \end{array}$$

Now, we put


$$\begin{array}{c} \psi_{n}=\frac{\varsigma^{2(n+1)}\prod_{j=0}^{n}\left[1-\left\{\rho_{j}+\xi_{j}+\lambda_{j}-\left(\xi_{j}\lambda_{j}+\left[\rho_{j}\lambda_{j}+\rho_{j}\xi_{j}\right](1-\varsigma )\right)-\rho_{j}\xi_{j}\lambda_{j}(1-\varsigma {)}^{2}\right\}(1-\varsigma )\right]}{\prod_{j=0}^{n}\left(1-\rho_{j}(1+\varsigma )\right)}, \end{array}$$

$$\begin{array}{c} \varphi_{n}=\frac{\varsigma^{2(n+1)}\prod_{j=0}^{n}\left[1-\left\{\rho_{j}+\xi_{j}+\lambda_{j}-\left(\xi_{j}\lambda_{j}+\left[\rho_{j}\lambda_{j}+\rho_{j}\xi_{j}\right](1-\varsigma )\right)-\rho_{j}\xi_{j}\lambda_{j}(1-\varsigma {)}^{2}\right\}(1-\varsigma )\right]}{\varsigma^{\left(1+n\right)}\prod_{j=0}^{n}[1-\rho_{j}(1+\rho_{j}(1-\xi_{j}(1-\varsigma ))(1-\lambda_{j}(1-\varsigma )))]}, \end{array}$$

and


$$\begin{array}{c} {℧}_{n}=\frac{\varsigma^{2(n+1)}\prod_{j=0}^{n}\left[1-\left\{\rho_{j}+\xi_{j}+\lambda_{j}-\left(\xi_{j}\lambda_{j}+\left[\rho_{j}\lambda_{j}+\rho_{j}\xi_{j}\right](1-\varsigma )\right)-\rho_{j}\xi_{j}\lambda_{j}(1-\varsigma {)}^{2}\right\}(1-\varsigma )\right]}{\prod_{j=0}^{n}[1-\rho_{j}(1+\varsigma )]}, \end{array}$$

then by the ratio test, we can write


$$\begin{array}{cc} & \frac{\psi_{n+1}}{\psi_{n}}\\ & \;=\frac{\varsigma^{2}\left[1-\left\{\begin{array}{c} \rho_{n+1}+\xi_{n+1}+\lambda_{n+1}-\left(\xi_{n+1}\lambda_{n+1}+\left[\rho_{n+1}\lambda_{n+1}+\rho_{n+1}\xi_{n+1}\right](1-\varsigma )\right)\\ -\rho_{n+1}\xi_{n+1}\lambda_{n+1}(1-\varsigma {)}^{2} \end{array}\right\}(1-\varsigma )\right]}{\left(1-\rho_{n+1}(1+\varsigma )\right)}, \end{array}$$

$$\begin{array}{cc} & \frac{\varphi_{n+1}}{\varphi_{n}}\\ & \;=\frac{\varsigma \left[1-\left\{\begin{array}{c} \rho_{n+1}+\xi_{n+1}+\lambda_{n+1}-\left(\xi_{n+1}\lambda_{n+1}+\left[\rho_{n+1}\lambda_{n+1}+\rho_{n+1}\xi_{n+1}\right](1-\varsigma )\right)\\ -\rho_{n+1}\xi_{n+1}\lambda_{n+1}(1-\varsigma {)}^{2} \end{array}\right\}(1-\varsigma )\right]}{\left[1-\rho_{n+1}(1+\rho_{n+1j}(1-\xi_{n+1}(1-\varsigma ))(1-\lambda_{n+1}(1-\varsigma )))\right]}, \end{array}$$

and


$$\begin{array}{cc} & \frac{{℧}_{n+1}}{{℧}_{n}}\\ & \;=\frac{\varsigma^{2}\left[1-\left\{\begin{array}{c} \rho_{n+1}+\xi_{n+1}+\lambda_{n+1}-\left(\xi_{n+1}\lambda_{n+1}+\left[\rho_{n+1}\lambda_{n+1}+\rho_{n+1}\xi_{n+1}\right](1-\varsigma )\right)\\ -\rho_{n+1}\xi_{n+1}\lambda_{n+1}(1-\varsigma {)}^{2} \end{array}\right\}(1-\varsigma )\right]}{\left[1-\rho_{n+1}(1+\varsigma )\right]}. \end{array}$$

Under the conditions $\lim_{n\to \infty }\rho_{n}=0,$ $\lim_{n\to \infty }\xi_{n}=0,$ and $\lim_{n\to \infty }\lambda_{n}=0$, we derive


$$\begin{array}{cc} \ell_{1} & =\lim_{n\to \infty }\frac{\psi_{n+1}}{\psi_{n}}=\varsigma^{2}<\varsigma <1,\\ \ell_{2} & =\lim_{n\to \infty }\frac{\varphi_{n+1}}{\varphi_{n}}=\varsigma <1,\\ \text{and }\ell_{3} & =\lim_{n\to \infty }\frac{{℧}_{n+1}}{{℧}_{n}}=\varsigma^{2}<\varsigma <1. \end{array}$$

The fact that $\ell_{1},\ell_{2},\ell_{3}<1$ allows us to apply the ratio test, confirming the convergence of the series $\sum_{n=0}^{\infty }\psi_{n},$ $\sum_{n=0}^{\infty }\varphi_{n}$ and $\sum_{n=0}^{\infty }{℧}_{n}$. From the convergence of these series, it necessarily follows that $\lim_{n\to \infty }\psi_{n}=0,$ $\lim_{n\to \infty }\varphi_{n}=0,$ and $\lim_{n\to \infty }{℧}_{n}=0$. Hence,


$$\begin{array}{c} \lim_{n\to \infty }\frac{\left\|\omega_{n+1}-\eta \right\|}{\left\|\varpi_{n+1}-\eta \right\|}=\lim_{n\to \infty }\frac{\left\|\omega_{n+1}-\eta \right\|}{\left\|s_{n+1}-\eta \right\|}=\lim_{n\to \infty }\frac{\left\|\omega_{n+1}-\eta \right\|}{\left\|\vartheta_{n+1}-\eta \right\|}=0. \end{array}$$

Therefore, based on Definition 1.7, we can analytically conclude that $\left\{\omega_{n}\right\}$ achieves convergence to a FP η at a faster rate than $\left\{\varpi_{n}\right\}$, $\left\{s_{n}\right\}$, and $\left\{\vartheta_{n}\right\}$. A distinct approach is utilized to demonstrate the faster convergence rate of $\left\{g_{n}\right\}$ and $\left\{\varrho_{n}\right\}$. By setting $\omega_{0}=g_{0}=\varrho_{0}$ and denoting the right-hand sides of (35), (39) and (40) as *b*_*n*_, *c*_*n*_, and *d*_*n*_, respectively, we obtain that


$$\begin{array}{cc} \frac{b_{n}}{c_{n}} & =\frac{\varsigma^{2(n+1)}\prod_{j=0}^{n}\left[1-\left\{\rho_{j}+\xi_{j}+\lambda_{j}-\left(\xi_{j}\lambda_{j}+\left[\rho_{j}\lambda_{j}+\rho_{j}\xi_{j}\right](1-\varsigma )\right)-\rho_{j}\xi_{j}\lambda_{j}(1-\varsigma {)}^{2}\right\}(1-\varsigma )\right]}{\varsigma^{\left(1+n\right)}\prod_{j=0}^{n}[1-\rho_{j}\xi_{j}\lambda_{j}(1-\varsigma )]},\\ & \to 0\text{ as }n\to \infty , \end{array}$$

and


$$\begin{array}{cc} \frac{b_{n}}{d_{n}} & =\frac{\varsigma^{2(n+1)}\prod_{j=0}^{n}\left[1-\left\{\rho_{j}+\xi_{j}+\lambda_{j}-\left(\xi_{j}\lambda_{j}+\left[\rho_{j}\lambda_{j}+\rho_{j}\xi_{j}\right](1-\varsigma )\right)-\rho_{j}\xi_{j}\lambda_{j}(1-\varsigma {)}^{2}\right\}(1-\varsigma )\right]}{\varsigma^{\left(1+n\right)}\prod_{j=0}^{n}[1-\lambda_{j}(1-\varsigma )]\left\|\varrho_{0}-\eta \right\|}\\ & \to 0\text{ as }n\to \infty . \end{array}$$

In accordance with Definition 1.6, it is concluded that the sequence $\left\{\omega_{n}\right\}$ converges to a FP η at a faster rate than sequences $\left\{g_{n}\right\}$ and $\left\{\varrho_{n}\right\}$ analytically.

## 4 Illustrative examples

In this section, we present illustration examples to validate our results.

**Example 1.** *Consider the BS* $\left(\ell^{2},{\left\|.\right\|}_{2}\right)$ *of square-summable sequences, equipped with its usual norm. Let* (*e*_*n*_) *denote the standard basis of* $\ell^{2}.$ *Describe*


$$\begin{array}{c} \Omega =\left\{\gamma e_{4}:\gamma \in [0,1]\right\}. \end{array}$$

*Define three mapping* ${ℜ}_{1},{ℜ}_{2},{ℜ}_{3}:\Omega \to \Omega$ *by*


$$\begin{array}{c} {ℜ}_{1}\left(\gamma e_{4}\right)=\left\{ \begin{array}{cc} \frac{e_{4}}{3}, & \gamma =1,\\ \frac{e_{4}}{8}, & otherwise, \end{array}\right. \end{array}$$

$$\begin{array}{c} {ℜ}_{2}\left(\gamma e_{4}\right)=\left\{ \begin{array}{cc} \frac{e_{4}}{9}, & \gamma =1,\\ \frac{e_{4}}{8}, & otherwise, \end{array}\right. \end{array}$$

*and*


$$\begin{array}{c} {ℜ}_{3}\left(\gamma e_{4}\right)=\left\{ \begin{array}{cc} \frac{e_{4}}{15}, & \gamma =1,\\ \frac{e_{4}}{8}, & otherwise. \end{array}\right. \end{array}$$

*First, we proceed to verify that* ${ℜ}_{1},$ ${ℜ}_{2}$ *and* ${ℜ}_{3}$ *are not NE. Fix* $\omega =e_{4}$ *and* $\upsilon =\frac{14}{15}e_{4},$ *then*


$$\begin{array}{cc} \left\|{ℜ}_{1}\omega -{ℜ}_{1}\upsilon \right\| & =\left\|{ℜ}_{1}\left(e_{4}\right)-{ℜ}_{1}\left(\frac{14}{15}e_{4}\right)\right\|\\ & =\left\|\frac{e_{4}}{3}-\frac{e_{4}}{8}\right\|=\frac{5}{24}>\frac{1}{15}=\left\|\omega -\upsilon \right\|. \end{array}$$

*Again, take* $\omega =e_{4}$ *and* $\upsilon =\frac{49}{50}e_{4},$ *then*


$$\begin{array}{cc} \left\|{ℜ}_{2}\omega -{ℜ}_{2}\upsilon \right\| & =\left\|{ℜ}_{2}\left(e_{4}\right)-{ℜ}_{2}\left(\frac{49}{50}e_{4}\right)\right\|\\ & =\left\|\frac{e_{4}}{15}-\frac{e_{4}}{8}\right\|=\frac{1}{72}>\frac{1}{50}=\left\|\omega -\upsilon \right\|. \end{array}$$

*Also, take* $\omega =e_{4}$ *and* $\upsilon =\frac{99}{100}e_{4},$ *then*


$$\begin{array}{cc} \left\|{ℜ}_{3}\omega -{ℜ}_{3}\upsilon \right\| & =\left\|{ℜ}_{3}\left(e_{4}\right)-{ℜ}_{3}\left(\frac{99}{100}e_{4}\right)\right\|\\ & =\left\|\frac{e_{4}}{9}-\frac{e_{4}}{8}\right\|=\frac{7}{120}>\frac{1}{100}=\left\|\omega -\upsilon \right\|. \end{array}$$

*Thus, having established that* ${ℜ}_{1},$ ${ℜ}_{2}$ *and* ${ℜ}_{3}$ *are not NE.*

*Next, we demonstrate that* ${ℜ}_{1},$ ${ℜ}_{2}$ *and* ${ℜ}_{3}$ *are* C−α−*NE mappings. We consider the following two options for* ${ℜ}_{1}:$

*Op. 1: If* $\omega =\gamma_{1}e_{4},$ $\upsilon =e_{4},$ *and* $\gamma_{1}\in [0,1),$ *we have*


$$\begin{array}{cc} & \alpha {\left\|{ℜ}_{1}\omega -\upsilon \right\|}^{2}+\alpha {\left\|{ℜ}_{1}\upsilon -\omega \right\|}^{2}+(1-2\alpha ){\left\|\omega -\upsilon \right\|}^{2}\\ & \;=\alpha {\left\|\frac{e_{4}}{8}-e_{4}\right\|}^{2}+\alpha {\left\|\frac{e_{4}}{3}-\gamma_{1}e_{4}\right\|}^{2}+(1-2\alpha ){\left\|\gamma_{1}e_{4}-e_{4}\right\|}^{2}\\ & \;=\frac{49}{64}\alpha +\alpha {\left(\frac{1}{3}-\gamma_{1}\right)}^{2}+(1-2\alpha ){\left(\gamma_{1}-1\right)}^{2}\\ & \;\geq \frac{49}{128},\text{ for }\alpha =\frac{1}{2}\\ & \;>{\left(\frac{5}{24}\right)}^{2}={\left\|{ℜ}_{1}\omega -{ℜ}_{1}\upsilon \right\|}^{2}. \end{array}$$

*Hence,*


$$\begin{array}{c} {\left\|{ℜ}_{1}\omega -{ℜ}_{1}\upsilon \right\|}^{2}\leq \alpha {\left\|{ℜ}_{1}\omega -\upsilon \right\|}^{2}+\alpha {\left\|{ℜ}_{1}\upsilon -\omega \right\|}^{2}+(1-2\alpha ){\left\|\omega -\upsilon \right\|}^{2}, \end{array}$$

*is true for* $\alpha =\frac{1}{2}.$

*Op. 2: If* $\omega =\gamma_{1}e_{4},$ $\upsilon =\gamma_{2}e_{4},$ *and* $\gamma_{1},\gamma_{2}\in [0,1),$ *we have*


$$\begin{array}{cc} {\left\|{ℜ}_{1}\omega -{ℜ}_{1}\upsilon \right\|}^{2} & =0\\ & \leq \alpha {\left\|{ℜ}_{1}\omega -\upsilon \right\|}^{2}+\alpha {\left\|{ℜ}_{1}\upsilon -\omega \right\|}^{2}+(1-2\alpha ){\left\|\omega -\upsilon \right\|}^{2}, \end{array}$$

*this is true for* α∈[0,1)*, and consequently for* $\alpha =\frac{1}{2}$.

*The following two options concern* ${ℜ}_{2}:$

*Op. 3: If we take* $\omega =\gamma_{2}e_{4},$ $\upsilon =e_{4},$ *and* $\gamma_{1}\in [0,1),$ *we get*


$$\begin{array}{cc} & \alpha {\left\|{ℜ}_{2}\omega -\upsilon \right\|}^{2}+\alpha {\left\|{ℜ}_{2}\upsilon -\omega \right\|}^{2}+(1-2\alpha ){\left\|\omega -\upsilon \right\|}^{2}\\ & \;=\alpha {\left\|\frac{e_{4}}{8}-e_{4}\right\|}^{2}+\alpha {\left\|\frac{e_{4}}{9}-\gamma_{2}e_{4}\right\|}^{2}+(1-2\alpha ){\left\|\gamma_{2}e_{4}-e_{4}\right\|}^{2}\\ & \;=\frac{49}{64}\alpha +\alpha {\left(\frac{1}{9}-\gamma_{2}\right)}^{2}+(1-2\alpha ){\left(\gamma_{2}-1\right)}^{2}\\ & \;\geq \frac{49}{128},\text{ for }\alpha =\frac{1}{2}\\ & \;>{\left(\frac{1}{72}\right)}^{2}={\left\|{ℜ}_{2}\omega -{ℜ}_{2}\upsilon \right\|}^{2}. \end{array}$$

*Hence,*


$$\begin{array}{c} {\left\|{ℜ}_{2}\omega -{ℜ}_{2}\upsilon \right\|}^{2}\leq \alpha {\left\|{ℜ}_{2}\omega -\upsilon \right\|}^{2}+\alpha {\left\|{ℜ}_{2}\upsilon -\omega \right\|}^{2}+(1-2\alpha ){\left\|\omega -\upsilon \right\|}^{2}, \end{array}$$

*holds for* $\alpha =\frac{1}{2}.$

*Op. 4: If we take* $\omega =\gamma_{1}e_{4},$ $\upsilon =\gamma_{2}e_{4},$ *and* $\gamma_{1},\gamma_{2}\in [0,1).$ *This aligns with (Op. 2), as discussed previously.*

*The following two options concern* ${ℜ}_{3}$:

*Op. 5: If we set* $\omega =\gamma_{3}e_{4},$ $\upsilon =e_{4},$ *and* $\gamma_{3}\in [0,1),$ *we have*


$$\begin{array}{cc} & \alpha {\left\|{ℜ}_{3}\omega -\upsilon \right\|}^{2}+\alpha {\left\|{ℜ}_{3}\upsilon -\omega \right\|}^{2}+(1-2\alpha ){\left\|\omega -\upsilon \right\|}^{2}\\ & \;=\alpha {\left\|\frac{e_{4}}{8}-e_{4}\right\|}^{2}+\alpha {\left\|\frac{e_{4}}{15}-\gamma_{3}e_{4}\right\|}^{2}+(1-2\alpha ){\left\|\gamma_{3}e_{4}-e_{4}\right\|}^{2}\\ & \;=\frac{49}{64}\alpha +\alpha {\left(\frac{1}{15}-\gamma_{3}\right)}^{2}+(1-2\alpha ){\left(\gamma_{3}-1\right)}^{2}\\ & \;\geq \frac{49}{128},\text{ for }\alpha =\frac{1}{2}\\ & \;>{\left(\frac{7}{120}\right)}^{2}={\left\|{ℜ}_{3}\omega -{ℜ}_{3}\upsilon \right\|}^{2}. \end{array}$$

*Hence,*


$$\begin{array}{c} {\left\|{ℜ}_{3}\omega -{ℜ}_{3}\upsilon \right\|}^{2}\leq \alpha {\left\|{ℜ}_{3}\omega -\upsilon \right\|}^{2}+\alpha {\left\|{ℜ}_{3}\upsilon -\omega \right\|}^{2}+(1-2\alpha ){\left\|\omega -\upsilon \right\|}^{2}, \end{array}$$

*holds for* $\alpha =\frac{1}{2}.$

*Op. 6: If we set* $\omega =\gamma_{1}e_{4},$ $\upsilon =\gamma_{3}e_{4},$ *and* $\gamma_{1},\gamma_{3}\in [0,1).$ *This is similar to Op. 2, discussed above.*

*Therefore, the mappings* ${ℜ}_{1},$ ${ℜ}_{2}$ *and* ${ℜ}_{3}$ *are* $C-\frac{1}{2}-$*NE mappings. Furthermore, it is evident that* $fix\left({ℜ}_{1}\right)=fix\left({ℜ}_{2}\right)=fix\left({ℜ}_{3}\right)=\frac{e_{4}}{8}$*, and thus* ℵ≠∅. *Describe* h(ϑ)=ϑ*, which is nondecreasing, satisfies* h(0)=0 *and* h(ϑ)>0 *for* ϑ∈(0,∞)*. Then, we deduce that*


$$\begin{array}{cc} d\left(\omega ,ℵ\right) & =\inf\left\|\omega -\frac{e_{4}}{8}\right\|\\ & =\inf\left\|\left(0,0,0,\gamma_{1}-\frac{1}{8},0,0,\cdots \right)\right\|\\ & =\sqrt{{\left(\gamma_{1}-\frac{1}{8}\right)}^{2}}. \end{array}$$

*Consequently,*


$$\begin{array}{c} d\left(\omega ,ℵ\right)=\left\{ \begin{array}{cc} 0,\text{ \textbackslash{} \textbackslash{} \textbackslash{} \textbackslash{} \textbackslash{} \textbackslash{} \textbackslash{} \textbackslash{} \textbackslash{} \textbackslash{} \textbackslash{} \textbackslash{} \textbackslash{} \textbackslash{} \textbackslash{} \textbackslash{} } & \gamma_{1}=\frac{1}{8},\\ \sqrt{{\left(\gamma_{1}-\frac{1}{8}\right)}^{2}}, & otherwise. \end{array}\right. \end{array}$$

*Thus,*


$$\begin{array}{c} h\left(d\left(\omega ,ℵ\right)\right)=\left\{ \begin{array}{cc} 0,\text{ \textbackslash{} \textbackslash{} \textbackslash{} \textbackslash{} \textbackslash{} \textbackslash{} \textbackslash{} \textbackslash{} \textbackslash{} \textbackslash{} \textbackslash{} \textbackslash{} \textbackslash{} \textbackslash{} \textbackslash{} \textbackslash{} } & \gamma_{1}=\frac{1}{8},\\ \sqrt{{\left(\gamma_{1}-\frac{1}{8}\right)}^{2}}, & otherwise. \end{array}\right. \end{array}$$

*It is clear that*


$$\begin{array}{c} \left\|{ℜ}_{1}\omega -\omega \right\|=\left\{ \begin{array}{cc} \frac{2}{3},\text{ \textbackslash{} \textbackslash{} \textbackslash{} \textbackslash{} \textbackslash{} \textbackslash{} \textbackslash{} \textbackslash{} \textbackslash{} } & \gamma_{1}=1,\\ \left|\frac{1}{8}-\gamma_{1}\right|, & \gamma_{1}\neq 1, \end{array}\right. \end{array}$$

$$\begin{array}{c} \left\|{ℜ}_{2}\omega -\omega \right\|=\left\{ \begin{array}{cc} \frac{7}{9},\text{ \textbackslash{} \textbackslash{} \textbackslash{} \textbackslash{} \textbackslash{} \textbackslash{} \textbackslash{} \textbackslash{} \textbackslash{} } & \gamma_{1}=1,\\ \left|\frac{1}{8}-\gamma_{1}\right|, & \gamma_{1}\neq 1, \end{array}\right. \end{array}$$

*and*


$$\begin{array}{c} \left\|{ℜ}_{3}\omega -\omega \right\|=\left\{ \begin{array}{cc} \frac{14}{15},\text{ \textbackslash{} \textbackslash{} \textbackslash{} \textbackslash{} \textbackslash{} \textbackslash{} \textbackslash{} \textbackslash{} \textbackslash{} } & \gamma_{1}=1,\\ \left|\frac{1}{8}-\gamma_{1}\right|, & \gamma_{1}\neq 1, \end{array}\right. \end{array}$$

*which implies that*


$$\begin{array}{c} \max\left\{\left\|{ℜ}_{1}\omega -\omega \right\|,\left\|{ℜ}_{2}\omega -\omega \right\|,\left\|{ℜ}_{3}\omega -\omega \right\|\right\}\geq h\left(d\left(\omega ,ℵ\right)\right). \end{array}$$

*Therefore* ${ℜ}_{1},$ ${ℜ}_{2}$ *and* ${ℜ}_{3}$ *fulfill Condition* $(B^{\prime }).$ *Thus, all requirements of Theorem 2.7 are satisfied and the mappings have a common FP* $\frac{e_{4}}{8}.$

**Example 2.** *Consider* Ξ=ℝ *as a BS equipped with its standard norm and* Ω=[−2,∞). *We define the mappings* ${ℜ}_{1},{ℜ}_{2},{ℜ}_{3}:\Omega \to \Omega$ *as follows:*


$$\begin{array}{c} {ℜ}_{1}\left(\omega \right)=\left\{ \begin{array}{cc} \frac{\omega }{6}, & \omega \in [-2,\frac{1}{2}]\\ \frac{\omega }{7}, & otherwise, \end{array}\right. \end{array}$$

$$\begin{array}{c} {ℜ}_{2}\left(\omega \right)=\left\{ \begin{array}{cc} \frac{\omega }{7}, & \omega \in [-2,\frac{1}{2}]\\ \frac{\omega }{10}, & otherwise, \end{array}\right. \end{array}$$

*and*


$$\begin{array}{c} {ℜ}_{3}\left(\omega \right)=\left\{ \begin{array}{cc} \frac{\omega }{10}, & \omega \in [-2,\frac{1}{2}]\\ \frac{\omega }{12}, & otherwise. \end{array}\right. \end{array}$$

*It’s easy to see that both mappings,* ${ℜ}_{1},$ ${ℜ}_{2}$ *and* ${ℜ}_{3}$*, are not NE due to their discontinuity at* $\omega =\frac{1}{2}$*. Additionally, similar to the previous example’s calculations, it can be readily confirmed that both* ${ℜ}_{1},$ ${ℜ}_{2}$ *and* ${ℜ}_{3}$ *are* C−α−NE *for* $\alpha =\frac{1}{20}.$ *Clearly,* $fix\left({ℜ}_{1}\right)=fix\left({ℜ}_{2}\right)=fix\left({ℜ}_{3}\right)=0,$ *this claim that* $ℵ=fix\left({ℜ}_{1}\right)\cap fix\left({ℜ}_{2}\right)\cap fix\left({ℜ}_{3}\right)\neq \emptyset .$ *Consider the nondecreasing function* $h\left(\vartheta \right)=\frac{\vartheta }{4}$ *with* h(0)=0 *and* h(ϑ)>0 *for* ϑ∈(0,∞)*. Consequently, we have*


$$\begin{array}{c} d\left(\omega ,ℵ\right)=\inf\left\|\omega -\eta \right\|=\inf\left\|\omega -0\right\|=\inf\left\|\omega \right\|. \end{array}$$

*Obviously*


$$\begin{array}{c} d\left(\omega ,ℵ\right)=\left\{ \begin{array}{cc} 0, & \omega \in [-2,\frac{1}{2}],\\ \frac{1}{2}, & otherwise, \end{array}\right. \end{array}$$

*and*


$$\begin{array}{c} h\left(d\left(\omega ,ℵ\right)\right)=\left\{ \begin{array}{cc} 0, & \omega \in [-2,\frac{1}{2}],\\ \frac{1}{8}, & otherwise. \end{array}\right. \end{array}$$

*Now, we discuss the following cases:*

*Ca 1: If* $\omega \in [-2,\frac{1}{2}],$ *we have*


$$\begin{array}{c} \left\|{ℜ}_{1}\omega -\omega \right\|=\left|\frac{\omega }{6}-\omega \right|=\left|\frac{5\omega }{6}\right| \end{array}$$

$$\begin{array}{c} \left\|{ℜ}_{2}\omega -\omega \right\|=\left|\frac{\omega }{7}-\omega \right|=\left|\frac{6\omega }{7}\right|, \end{array}$$

*and*


$$\begin{array}{c} \left\|{ℜ}_{3}\omega -\omega \right\|=\left|\frac{\omega }{10}-\omega \right|=\left|\frac{9\omega }{10}\right|. \end{array}$$

*Hence*


$$\begin{array}{c} \max\left\{\left\|{ℜ}_{1}\omega -\omega \right\|,\left\|{ℜ}_{2}\omega -\omega \right\|,\left\|{ℜ}_{3}\omega -\omega \right\|\right\}\geq h\left(d\left(\omega ,ℵ\right)\right). \end{array}$$

*Ca. 2: If* $\omega \in \left(\frac{1}{2},\infty \right),$ *one has*


$$\begin{array}{c} \left\|{ℜ}_{1}\omega -\omega \right\|=\left|\frac{\omega }{7}-\omega \right|=\left|\frac{6\omega }{7}\right|, \end{array}$$

$$\begin{array}{c} \left\|{ℜ}_{2}\omega -\omega \right\|=\left|\frac{\omega }{10}-\omega \right|=\left|\frac{9\omega }{10}\right|, \end{array}$$

*and*


$$\begin{array}{c} \left\|{ℜ}_{3}\omega -\omega \right\|=\left|\frac{\omega }{12}-\omega \right|=\left|\frac{11\omega }{12}\right|. \end{array}$$

*Hence*


$$\begin{array}{c} \max\left\{\left\|{ℜ}_{1}\omega -\omega \right\|,\left\|{ℜ}_{2}\omega -\omega \right\|,\left\|{ℜ}_{3}\omega -\omega \right\|\right\}\geq h\left(d\left(\omega ,ℵ\right)\right). \end{array}$$

*Therefore, both* ${ℜ}_{1},$ ${ℜ}_{2}$ *and* ${ℜ}_{3}$ *satisfy Condition* $\left(B^{\prime }\right)$*, meaning the hypotheses of Theorem 2.7 are fulfilled. Thus, both mappings share the common FP of* ω=0.

## 5 Numerical results

In this section, we assess the numerical performance of the proposed iterative scheme (7), hereafter referred to as the **Proposed MZI Method**, through a series of computational experiments. The method is applied in both scalar and Hilbert space frameworks, specifically using Examples 4.1 and 4.2. For comparative analysis, we implement and evaluate the following five established iterative schemes:

- the Noor iteration scheme (27) (**Noor**),
- the S-iteration scheme (28) (**S-Iteration**),
- the Ishikawa iteration scheme (29) (**Ishikawa**),
- the Abbas–Nazir iteration scheme (30) (**Abbas–Nazir**),
- the HR iteration scheme (31) (**HR**).

For each method, unless explicitly stated otherwise, the initial point and associated control parameters namely the sequences $\left\{\rho_{n}\right\}$, $\left\{\xi_{n}\right\}$, and $\left\{\lambda_{n}\right\}$ are randomly selected from the open interval (0,1). The experimental results highlight the efficiency and enhanced convergence characteristics of the proposed method in comparison with the aforementioned algorithms.

### 5.1 Experiment 1

This experiment is carried out using Example 4.1, which is defined in the infinite-dimensional Hilbert space $\ell^{2}$, with the feasible set restricted to scalar multiples of the standard basis vector *e*_4_. The objective is to evaluate the numerical performance of the **Proposed MZI Method** in comparison with five classical iterative schemes: Noor, S-Iteration, Ishikawa, Abbas–Nazir, and HR. To ensure a comprehensive and unbiased comparison, each method is tested under six distinct initial conditions of the form $\omega_{0}=\gamma_{0}e_{4}$, where $\gamma_{0}\in [0,1]$ is chosen to span representative values. These include the FP, boundary values, and intermediate scalars, thereby enabling a detailed examination of each method’s sensitivity to the initial guess.

For every initial point, we record both the number of iterations required to achieve convergence (up to a specified tolerance of 10^−9^) and the corresponding CPU time measured in seconds. This experimental setup enables a detailed comparison of convergence speed and computational efficiency across all algorithms.

The six initial values used in Experiment 1 are given by


$$\begin{array}{c} \omega_{0}=\gamma_{0}e_{4},\;\text{where }\gamma_{0}\in \left\{\frac{1}{10},\;\frac{1}{6},\;0.25,\;0.5,\;0.75,\;1\right\}. \end{array}$$

#### 5.1.1 Interpretation of results.

**Fig 1:**
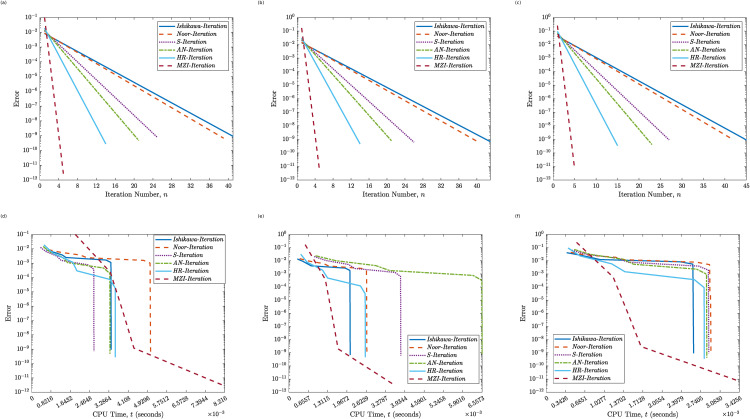
Convergence behavior of the Proposed MZI Method and five classical methods for Example 4.1. Panels **(a)**–**(c)** show the error versus the iteration count (*k*) for $\gamma_{0}=\frac{1}{10}$, $\gamma_{0}=\frac{1}{6}$, and $\gamma_{0}=0.25$, respectively. Panels **(d)**–**(f)** show the error versus CPU time (*t*) for $\gamma_{0}=\frac{1}{10}$, $\gamma_{0}=\frac{1}{6}$, and $\gamma_{0}=0.25$, respectively.

**Fig 2:**
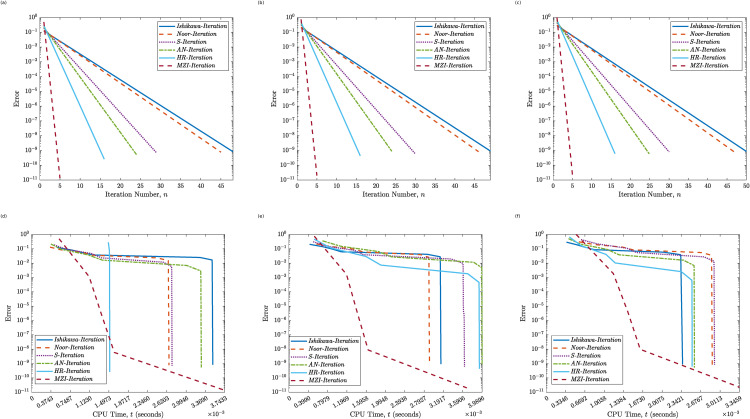
Convergence behavior of the Proposed MZI Method and five classical methods for Example 4.1. Panels **(a)**–**(c)** show the error versus the iteration count (*k*) for $\gamma_{0}=0.5$, $\gamma_{0}=0.75$, and $\gamma_{0}=1$, respectively. Panels **(d)**–**(f)** show the error versus CPU time (*t*) for $\gamma_{0}=0.5$, $\gamma_{0}=0.75$, and $\gamma_{0}=1$, respectively.

Table 1, together with Figs 1 and 2, presents a comparative analysis of the convergence behavior and computational efficiency of the proposed MZI method relative to five well-known iterative schemes: Ishikawa, Noor, S-Iteration, Abbas–Nazir (AN), and HR. The performance is assessed using six representative initial values $\gamma_{0}\in \left\{\frac{1}{10},\frac{1}{6},0.25,0.5,0.75,1\right\}$.

**Table 1:** Performance of the proposed MZI method vs. five classical methods for Example 4.1 using six initial points. Iterations and CPU times correspond to Figs 1 and 2.

Initial Point $\gamma_{0}$	Ishikawa-Iteration		Noor-Iteration		S-Iteration		AN-Iteration		HR-Iteration		MZI-Iteration	
	Iter	Time (s)	Iter	Time (s)	Iter	Time (s)	Iter	Time (s)	Iter	Time (s)	Iter	Time (s)
$\frac{1}{10}$	41	0.0003	39	0.0053	25	0.0029	21	0.0036	14	0.0038	5	0.0090
$\frac{1}{6}$	43	0.0023	40	0.0029	26	0.0041	21	0.0069	14	0.0028	5	0.0039
0.25	45	0.0029	42	0.0032	27	0.0031	23	0.0031	15	0.0030	5	0.0038
0.5	48	0.0038	45	0.0029	29	0.0030	24	0.0036	16	0.0018	5	0.0042
0.75	49	0.0035	46	0.0031	30	0.0039	24	0.0042	16	0.0042	5	0.0041
1	50	0.0028	47	0.0032	30	0.0032	25	0.0028	16	0.0028	5	0.0037

A clear and consistent trend is observed across all methods: as the value of $\gamma_{0}$ increases, both the iteration count and the computational time generally increase. Among the classical methods, the Ishikawa and Noor schemes exhibit the slowest convergence, requiring between 41 and 50 iterations, and 39–47 iterations, respectively. In contrast, the proposed MZI method achieves significantly faster convergence, completing in only 5 iterations across all initial values tested. While the HR method demonstrates a notable improvement over the other classical schemes, requiring between 14 and 16 iterations, it still remains outperformed by the MZI approach.

In terms of CPU time, the MZI method maintains consistently low execution times always below 0.009 seconds despite its superior convergence properties. For smaller initial values (e.g., $\gamma_{0}=\frac{1}{10}$ and $\frac{1}{6}$), its advantage in both speed and efficiency becomes even more pronounced. Although the AN and HR methods occasionally exhibit moderate improvements over the Noor and Ishikawa schemes, they are neither as stable nor as efficient as the proposed MZI method.

Overall, the numerical findings strongly support the robustness and efficiency of the proposed MZI method. Its independence from the initial condition, rapid convergence, and minimal computational overhead emphasize its effectiveness compared to conventional fixed-point iterative schemes, particularly in infinite-dimensional Hilbert space settings.

### 5.2 Experiment 2

This experiment is based on Example 4.2, which is defined on the real BS ℝ over the domain Ω=[−2,∞). The operator involved is piecewise-defined and exhibits discontinuity at $\omega =\frac{1}{2}$, making the convergence dynamics of iterative methods particularly noteworthy. Similar to Experiment 1, the performance of the proposed **MZI Method** is compared against five classical iterative schemes: Noor, S-Iteration, Ishikawa, Abbas–Nazir (AN), and HR.

To ensure a comprehensive evaluation, each method is tested using six distinct initial points selected to span various regions of the domain including negative values, points near the discontinuity, and values in the positive range. This choice facilitates analysis of each method’s robustness, sensitivity to initialization, and efficiency in the presence of operator non-smoothness. For each initial point, the number of iterations required for convergence (with tolerance 10^−6^) and the corresponding CPU execution time (in seconds) are recorded.

The initial values used in Experiment 2 are given by:


$$\begin{array}{c} \omega_{0}\in \left\{-1.5,\;0,\;0.25,\;0.5,\;1,\;2\right\}. \end{array}$$

#### 5.2.1 Interpretation of results.

**Fig 3:**
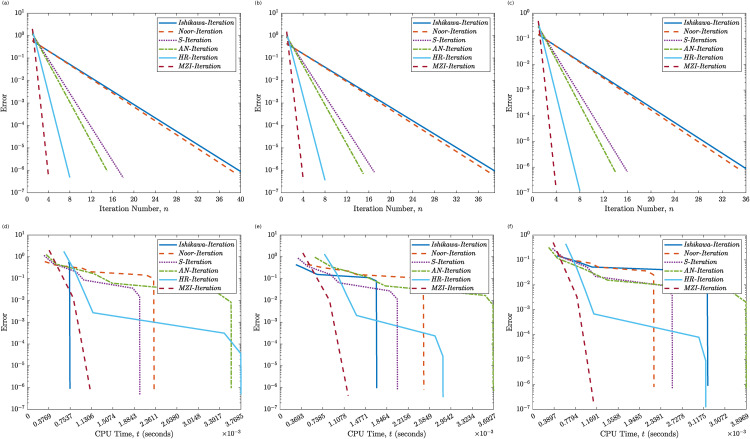
Convergence behavior of the Proposed MZI Method versus five classical methods for Example 4.2. Panels **(a)–(c)** show the error versus the iteration count (*k*) for $\omega_{0}=-2.00$, $\omega_{0}=-1.50$, and $\omega_{0}=-0.50$, respectively. Panels **(d)–(f)** show the error versus CPU time (*t*) for $\omega_{0}=-2.00$, $\omega_{0}=-1.50$, and $\omega_{0}=-0.50$, respectively.

**Fig 4:**
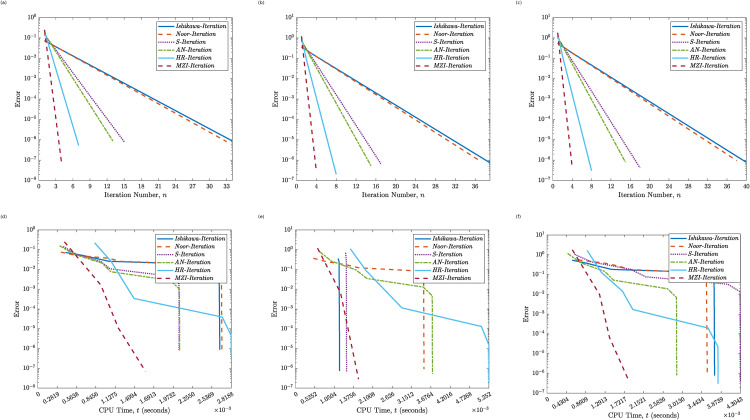
Convergence of the Proposed MZI Method and five classical methods for Example 4.2. Panels **(a)–(c)** show the error versus the iteration count (*k*) for $\omega_{0}=0.25$, $\omega_{0}=1.20$, and $\omega_{0}=1.80$, respectively. Panels **(d)–(f)** show the error versus CPU time (*t*) for $\omega_{0}=0.25$, $\omega_{0}=1.20$, and $\omega_{0}=1.80$, respectively.

Table 2, supported by Figs 3 and 4, presents a comparative analysis of six iterative schemes applied to Example 4.2, where the operator is piecewise-defined and non-smooth. The results highlight trends in iteration count and CPU time across initial points $\omega_{0}\in \{-2.00,\;-1.50,\;-0.50,\;0.25,\;1.20,\;1.80\}$.

**Table 2:** Comparison of iteration counts and execution times for the Proposed MZI Method and five classical methods on Example 4.2, using six initial points. Results correspond to Figs 3 and 4.

Initial Point $\omega_{0}$	Ishikawa-Iteration		Noor-Iteration		S-Iteration		AN-Iteration		HR-Iteration		MZI-Iteration	
	Iter	Time (s)	Iter	Time (s)	Iter	Time (s)	Iter	Time (s)	Iter	Time (s)	Iter	Time (s)
–2.00	40	0.0009	39	0.0024	18	0.0021	15	0.0040	8	0.0041	4	0.0013
–1.50	39	0.0018	38	0.0027	17	0.0022	15	0.0039	8	0.0030	4	0.0014
–0.50	36	0.0035	35	0.0024	16	0.0027	14	0.0043	8	0.0033	4	0.0013
0.25	34	0.0030	33	0.0030	15	0.0022	13	0.0022	7	0.0030	4	0.0019
1.20	39	0.0038	37	0.0037	17	0.0016	15	0.0040	8	0.0054	4	0.0019
1.80	40	0.0040	38	0.0003	18	0.0046	15	0.0031	8	0.0040	4	0.0003

Among the traditional methods, Ishikawa and Noor iterations show consistently higher iteration counts, ranging from 34 to 50, with increased computation times as the initial value approaches or exceeds the discontinuity at ω=0.5. The S-Iteration and AN methods demonstrate improved performance but still display sensitivity to the choice of $\omega_{0}$, particularly in regions near the discontinuity.

The HR method shows a substantial improvement over its predecessors, converging in 7–8 iterations across all cases and maintaining computation times within 0.003 to 0.005 seconds. However, the proposed MZI method outperforms all others by converging in just 4 iterations for every tested value of $\omega_{0}$. Furthermore, its CPU time remains remarkably low never exceeding 0.0019 seconds and as low as 0.0003 seconds in some cases. These findings confirm the robustness, speed, and efficiency of the MZI method, which maintains high performance irrespective of operator non-smoothness or the selected initial point. Its insensitivity to initial conditions and superior convergence properties highlight its practical advantage over existing methods in solving nonlinear problems in BSs.

## 6 Supportive application

This section utilizes the obtained results to examine the existence of solutions for a general class of VFIEs with deviating argument in a BS. We first present the MZI for a single mapping:


$$\begin{array}{c} \left\{ \begin{array}{c} \omega_{0}\in \Omega ,\\ \omega_{n+1}=ℜ\nu_{n},\\ \nu_{n}=\left(1-\rho_{n}\right)\tau_{n}+\rho_{n}ℜ\tau_{n},\\ \tau_{n}=\left(1-\xi_{n}\right)\kappa_{n}+\xi_{n}ℜ\kappa_{n},\\ \kappa_{n}=ℜ\left[\left(1-\lambda_{n}\right)\omega_{n}+\lambda_{n}ℜ\omega_{n}\right], \end{array}\right. \end{array}$$ (41)

Our starting point is the general nonlinear VFIEs with a deviating argument in BSs, originally introduced by Lungu and Rus [39]:


$$\begin{array}{c} \varkappa \left(\zeta ,\delta \right)=\Psi \left(\zeta ,\delta ,z\left(\varkappa \left(\zeta ,\delta \right)\right)\right)+\int_{0}^{\zeta }\int_{0}^{\delta }ℑ\left(\zeta ,\delta ,b,c,\varkappa \left(b,c\right)\right)dbdc, \end{array}$$ (42)

for all $\zeta ,\delta \in {\mathbb{R}}_{+}.$ For any BS (Ξ,‖.‖) and ℏ>0, it is possible to define


$$\begin{array}{c} \Theta_{\hbar }=\left\{\varkappa \in C\left({\mathbb{R}}_{+}^{2},\Xi \right):\text{ there exists }N\left(\varkappa \right)>0\text{ such that }\left|\varkappa \left(\zeta ,\delta \right)\right|e^{-\hbar \left(\zeta +\delta \right)}\leq N\left(\varkappa \right)\right\}. \end{array}$$

Considering Bielecki’s norm [40] on $\Theta_{\hbar }$ as


$$\begin{array}{c} {\left\|\varkappa \right\|}_{\hbar }=\sup_{\zeta ,\delta \in {\mathbb{R}}_{+}}\left|\varkappa \left(\zeta ,\delta \right)\right|e^{-\hbar \left(\zeta +\delta \right)}. \end{array}$$

It is known that $\left(\Theta_{\hbar },{\left\|.\right\|}_{\hbar }\right)$ forms a BS.

Now, the MZI (41) will be considered under the following assertions:

(A_1_) $\Psi \in C\left({\mathbb{R}}_{+}^{2}\times \Xi ,\Xi \right)$ and $ℑ\in C\left({\mathbb{R}}_{+}^{4}\times \Xi ,\Xi \right),$

(A_2_) $z:\Theta_{\hbar }\to \Theta_{\hbar }$ is such that there is *M*_*z*_ > 0 such that


$$\begin{array}{c} \left|z\left(\varkappa \left(\zeta ,\delta \right)\right)-z\left(\tilde{\varkappa }\left(\zeta ,\delta \right)\right)\right|\leq M_{z}{\left\|\varkappa -\tilde{\varkappa }\right\|}_{\hbar }e^{\hbar \left(\zeta +\delta \right)}, \end{array}$$

for all $\zeta ,\delta \in {\mathbb{R}}_{+}$ and $\varkappa ,\tilde{\varkappa }\in \Theta_{\hbar },$

(A_3_) for all $\zeta ,\delta \in {\mathbb{R}}_{+}$ and $\rho_{1},\rho_{2}\in \Xi ,$ there exists $M_{\Psi }>0$ such that


$$\begin{array}{c} \left|\Psi \left(\zeta ,\delta ,\rho_{1}\right)-\Psi \left(\zeta ,\delta ,\rho_{2}\right)\right|\leq M_{\Psi }\left|\rho_{1}-\rho_{2}\right|, \end{array}$$

(A_4_) for all $\zeta ,\delta ,b,c\in {\mathbb{R}}_{+}$ and $\rho_{1},\rho_{2}\in \Xi ,$ there exists $M_{ℑ}>0$ such that


$$\begin{array}{c} \left|ℑ\left(\zeta ,\delta ,b,c,\rho_{1}\right)-ℑ\left(\zeta ,\delta ,b,c,\rho_{2}\right)\right|\leq M_{ℑ}\left(\zeta ,\delta ,b,c\right)\left|\rho_{1}-\rho_{2}\right|, \end{array}$$

(A_5_) for all $\zeta ,\delta \in {\mathbb{R}}_{+},$ there exists *M* > 0 such that $\int_{0}^{\zeta }\int_{0}^{\delta }M_{ℑ}\left(\zeta ,\delta ,b,c\right)e^{\hbar \left(b+c\right)}dbdc\leq Me^{\hbar \left(\zeta +\delta \right)},$ where $M_{ℑ}\in C\left({\mathbb{R}}_{+}^{4},{\mathbb{R}}_{+}\right),$

(A_6_) $M_{\Psi }M_{z}+M<1.$

Moreover, the integral equation (42) has a unique solution $\tilde{\varkappa }\in \Theta_{\hbar }$, and the sequence of successive approximations


$$\begin{array}{c} \varkappa_{n+1}\left(\zeta ,\delta \right)=\Psi \left(\zeta ,\delta ,z\left(\varkappa_{n}\left(\zeta ,\delta \right)\right)\right)+\int_{0}^{\zeta }\int_{0}^{\delta }ℑ\left(\zeta ,\delta ,b,c,\varkappa_{n}\left(b,c\right)\right)dbdc, \end{array}$$ (43)

converges uniformly to $\tilde{\varkappa },$ for all n=0,1,2,⋯.

The forthcoming result provides an affirmative demonstration of the existence and uniqueness of the solution for such integral equations.

**Theorem 6.1.** *Assume that* $\left\{\omega_{n}\right\}$ *is a sequence produced by the MZI (41), with control sequences* $\{\rho_{n}\},$ $\left\{\xi_{n}\right\}$ *and* $\left\{\lambda_{n}\right\}$ *on (0,1) fulfilling* $\sum_{n=0}^{\infty }\rho_{n}=\infty ,$ $\sum_{n=0}^{\infty }\xi_{n}=\infty ,$ $\sum_{n=0}^{\infty }\lambda_{n}=\infty ,$ *and* $\sum_{n=0}^{\infty }\lambda_{n}\xi_{n}\rho_{n}=\infty$*. If the assertions (A*_*1*_*)-(A*_*6*_*) and (43) are satisfied, then the VFIE (42) admits a unique solution* $\tilde{\varkappa }\in \Theta_{\hbar }$*, and the MZI (41) converges strongly to this solution.*

*Proof.* Assume the iterative sequence $\left\{\omega_{n}\right\}$ induced by the MZI (41). Describe and operator $ℜ:\Theta_{\hbar }\to \Theta_{\hbar }$ as


$$\begin{array}{c} ℜ\left(\varkappa \left(\zeta ,\delta \right)\right)=\Psi \left(\zeta ,\delta ,z\left(\varkappa \left(\zeta ,\delta \right)\right)\right)+\int_{0}^{\zeta }\int_{0}^{\delta }ℑ\left(\zeta ,\delta ,b,c,\varkappa \left(b,c\right)\right)dbdc. \end{array}$$

We shall prove that $\omega_{n}\to \tilde{\varkappa }$ as n→∞. Using the the iteration (41), we have


$$\begin{array}{cc} {\left\|\omega_{n+1}-\tilde{\varkappa }\right\|}_{\hbar } & ={\left\|ℜ\nu_{n}-ℜ\tilde{\varkappa }\right\|}_{\hbar }\\ & =\sup_{\zeta ,\delta \in {\mathbb{R}}_{+}}\left|ℜ\left(\nu_{n}\left(\zeta ,\delta \right)\right)-ℜ\left(\tilde{\varkappa }\left(\zeta ,\delta \right)\right)\right|e^{-\hbar \left(\zeta +\delta \right)}. \end{array}$$ (44)

Now,


$$\begin{array}{cc} & \left|ℜ\left(\nu_{n}\left(\zeta ,\delta \right)\right)-ℜ\left(\tilde{\varkappa }\left(\zeta ,\delta \right)\right)\right|\\ & \;\leq \left|\Psi \left(\zeta ,\delta ,z\left(\nu_{n}\left(\zeta ,\delta \right)\right)\right)-\Psi \left(\zeta ,\delta ,z\left(\tilde{\varkappa }\left(\zeta ,\delta \right)\right)\right)\right|\\ & \;+\left|\int_{0}^{\zeta }\int_{0}^{\delta }ℑ\left(\zeta ,\delta ,b,c,\nu_{n}\left(b,c\right)\right)dbdc-\int_{0}^{\zeta }\int_{0}^{\delta }ℑ\left(\zeta ,\delta ,b,c,\tilde{\varkappa }\left(b,c\right)\right)dbdc\right|\\ & \;\leq M_{\Psi }\left|z\left(\nu_{n}\left(\zeta ,\delta \right)\right)-z\left(\tilde{\varkappa }\left(\zeta ,\delta \right)\right)\right|\\ & \;+\int_{0}^{\zeta }\int_{0}^{\delta }\left|ℑ\left(\zeta ,\delta ,b,c,\nu_{n}\left(b,c\right)\right)-ℑ\left(\zeta ,\delta ,b,c,\tilde{\varkappa }\left(b,c\right)\right)\right|dbdc\\ & \;\leq M_{\Psi }M_{z}{\left\|\nu_{n}-\tilde{\varkappa }\right\|}_{\hbar }e^{\hbar \left(\zeta +\delta \right)}+\int_{0}^{\zeta }\int_{0}^{\delta }M_{ℑ}\left(\zeta ,\delta ,b,c\right)\left|\nu_{n}\left(b,c\right)-\tilde{\varkappa }\left(b,c\right)\right|dbdc\\ & \;\leq M_{\Psi }M_{z}{\left\|\nu_{n}-\tilde{\varkappa }\right\|}_{\hbar }e^{\hbar \left(\zeta +\delta \right)}+M{\left\|\nu_{n}-\tilde{\varkappa }\right\|}_{\hbar }e^{\hbar \left(\zeta +\delta \right)}\\ & \;=\left(M_{\Psi }M_{z}+M\right){\left\|\nu_{n}-\tilde{\varkappa }\right\|}_{\hbar }e^{\hbar \left(\zeta +\delta \right)}. \end{array}$$ (45)

Applying (45) in (44), we conclude that


$$\begin{array}{c} {\left\|\omega_{n+1}-\tilde{\varkappa }\right\|}_{\hbar }\leq \left(M_{\Psi }M_{z}+M\right){\left\|\nu_{n}-\tilde{\varkappa }\right\|}_{\hbar }. \end{array}$$ (46)

Again, utilizing the algorithm (41), we have


$$\begin{array}{cc} {\left\|\nu_{n}-\tilde{\varkappa }\right\|}_{\hbar } & ={\left\|\left(1-\rho_{n}\right)\tau_{n}+\rho_{n}ℜ\tau_{n}-ℜ\tilde{\varkappa }\right\|}_{\hbar }\\ & \leq \left(1-\rho_{n}\right){\left\|\tau_{n}-ℜ\tilde{\varkappa }\right\|}_{\hbar }+\rho_{n}{\left\|ℜ\tau_{n}-ℜ\tilde{\varkappa }\right\|}_{\hbar }. \end{array}$$ (47)

Similar to (44), we can write


$$\begin{array}{c} {\left\|ℜ\tau_{n}-ℜ\tilde{\varkappa }\right\|}_{\hbar }=\sup_{\zeta ,\delta \in {\mathbb{R}}_{+}}\left|ℜ\left(\tau_{n}\left(\zeta ,\delta \right)\right)-ℜ\left(\tilde{\varkappa }\left(\zeta ,\delta \right)\right)\right|e^{-\hbar \left(\zeta +\delta \right)}. \end{array}$$ (48)

Similar to (45), one has


$$\begin{array}{c} \left|ℜ\left(\tau_{n}\left(\zeta ,\delta \right)\right)-ℜ\left(\tilde{\varkappa }\left(\zeta ,\delta \right)\right)\right|\leq \left(M_{\Psi }M_{z}+M\right){\left\|\tau_{n}-\tilde{\varkappa }\right\|}_{\hbar }e^{\hbar \left(\zeta +\delta \right)}. \end{array}$$ (49)

From (49) in (48), we can write


$$\begin{array}{c} {\left\|ℜ\tau_{n}-ℜ\tilde{\varkappa }\right\|}_{\hbar }\leq \left(M_{\Psi }M_{z}+M\right){\left\|\tau_{n}-\tilde{\varkappa }\right\|}_{\hbar } \end{array}$$ (50)

Applying (50) in (47), we get


$$\begin{array}{cc} {\left\|\nu_{n}-\tilde{\varkappa }\right\|}_{\hbar } & \leq \left(1-\rho_{n}\right){\left\|\tau_{n}-\tilde{\varkappa }\right\|}_{\hbar }+\rho_{n}\left(M_{\Psi }M_{z}+M\right){\left\|\tau_{n}-\tilde{\varkappa }\right\|}_{\hbar }\\ & =\left[1-\rho_{n}(1-\left(M_{\Psi }M_{z}+M\right))\right]{\left\|\tau_{n}-\tilde{\varkappa }\right\|}_{\hbar }. \end{array}$$ (51)

Similarly, by the algorithm (41), we have


$$\begin{array}{cc} {\left\|\tau_{n}-\tilde{\varkappa }\right\|}_{\hbar } & \leq \left(1-\xi_{n}\right)\left\|\kappa_{n}-\tilde{\varkappa }\right\|+\xi_{n}\left(M_{\Psi }M_{z}+M\right){\left\|\kappa_{n}-\tilde{\varkappa }\right\|}_{\hbar }\\ & =\left[1-\xi_{n}\left(1-\left(M_{\Psi }M_{z}+M\right)\right)\right]{\left\|\kappa_{n}-\tilde{\varkappa }\right\|}_{\hbar }. \end{array}$$ (52)

From (52) in (51), one has


$$\begin{array}{c} {\left\|\nu_{n}-\tilde{\varkappa }\right\|}_{\hbar }\leq \left[1-\rho_{n}(1-\left(M_{\Psi }M_{z}+M\right))\right]\left[1-\xi_{n}\left(1-\left(M_{\Psi }M_{z}+M\right)\right)\right]{\left\|\kappa_{n}-\tilde{\varkappa }\right\|}_{\hbar }. \end{array}$$ (53)

Finally,


$$\begin{array}{cc} {\left\|\kappa_{n}-\tilde{\varkappa }\right\|}_{\hbar } & ={\left\|ℜ\left[\left(1-\lambda_{n}\right)\omega_{n}+\lambda_{n}ℜ\omega_{n}\right]-ℜ\tilde{\varkappa }\right\|}_{\hbar }\\ & \leq {\left\|\left(1-\lambda_{n}\right)\omega_{n}+\lambda_{n}ℜ\omega_{n}-\tilde{\varkappa }\right\|}_{\hbar }\\ & \leq \left(1-\lambda_{n}\right)\left\|\omega_{n}-\tilde{\varkappa }\right\|+\lambda_{n}{\left\|ℜ\omega_{n}-ℜ\tilde{\varkappa }\right\|}_{\hbar }\\ & \leq \left(1-\lambda_{n}\right)\left\|\omega_{n}-\tilde{\varkappa }\right\|+\lambda_{n}\left(M_{\Psi }M_{z}+M\right){\left\|\omega_{n}-\tilde{\varkappa }\right\|}_{\hbar }\\ & =\left[1-\lambda_{n}(1-\left(M_{\Psi }M_{z}+M\right))\right]{\left\|\omega_{n}-\tilde{\varkappa }\right\|}_{\hbar }. \end{array}$$ (54)

Applying (54) in (53), we have


$$\begin{array}{cc} {\left\|\nu_{n}-\tilde{\varkappa }\right\|}_{\hbar } & \leq \left[1-\rho_{n}(1-\left(M_{\Psi }M_{z}+M\right))\right]\left[1-\xi_{n}\left(1-\left(M_{\Psi }M_{z}+M\right)\right)\right]\\ & \left[1-\lambda_{n}(1-\left(M_{\Psi }M_{z}+M\right))\right]{\left\|\omega_{n}-\tilde{\varkappa }\right\|}_{\hbar }. \end{array}$$ (55)

Reflecting (55) in (46), we obtain that


$$\begin{array}{cc} & {\left\|\omega_{n+1}-\tilde{\varkappa }\right\|}_{\hbar }\\ & \;\leq {\left(M_{\Psi }M_{z}+M\right)}^{2}\{1-[\rho_{n}+\xi_{n}+\lambda_{n}-\left(\xi_{n}\lambda_{n}+\left[\rho_{n}\lambda_{n}+\rho_{n}\xi_{n}\right](1-\left(M_{\Psi }M_{z}+M\right))\right)\\ & \;-\rho_{n}\xi_{n}\lambda_{n}\left(1-{\left(M_{\Psi }M_{z}+M\right)}^{2}\right)]\left(1-\left(M_{\Psi }M_{z}+M\right)\right)\}{\left\|\omega_{n}-\tilde{\varkappa }\right\|}_{\hbar }. \end{array}$$ (56)

For simplicity, we consider


$$\begin{array}{cc} {℘}_{n} & =[\rho_{n}+\xi_{n}+\lambda_{n}-\left(\xi_{n}\lambda_{n}+\left[\rho_{n}\lambda_{n}+\rho_{n}\xi_{n}\right](1-\left(M_{\Psi }M_{z}+M\right))\right)\\ & \;-\rho_{n}\xi_{n}\lambda_{n}\left(1-{\left(M_{\Psi }M_{z}+M\right)}^{2}\right)], \end{array}$$

such that ${℘}_{n}\in (0,1)$. Since $\sum_{n=0}^{\infty }\rho_{n}=\infty ,$ $\sum_{n=0}^{\infty }\xi_{n}=\infty ,$ $\sum_{n=0}^{\infty }\lambda_{n}=\infty ,$ and $\sum_{n=0}^{\infty }\lambda_{n}\xi_{n}\rho_{n}=\infty ,$ we have $\sum_{n=0}^{\infty }{℘}_{n}=\infty .$

Further, since $M_{\Psi }M_{z}+M<1$, the inequality (56) reduces to


$$\begin{array}{cc} {\left\|\omega_{n+1}-\tilde{\varkappa }\right\|}_{\hbar } & \leq \left[\left(1-{℘}_{n}\right)\left(1-\left(M_{\Psi }M_{z}+M\right)\right)\right]{\left\|\omega_{n}-\tilde{\varkappa }\right\|}_{\hbar }.\\ & =\left(1-\left[{℘}_{n}\left(1-\left(M_{\Psi }M_{z}+M\right)\right)-\left(M_{\Psi }M_{z}+M\right)\right]\right){\left\|\omega_{n}-\tilde{\varkappa }\right\|}_{\hbar }. \end{array}$$

Following the same procedure, we get


$$\begin{array}{cc} {\left\|\omega_{n}-\tilde{\varkappa }\right\|}_{\hbar } & \leq \left(1-\left[{℘}_{n-1}\left(1-\left(M_{\Psi }M_{z}+M\right)\right)-\left(M_{\Psi }M_{z}+M\right)\right]\right){\left\|\omega_{n-1}-\tilde{\varkappa }\right\|}_{\hbar }\\ {\left\|\omega_{n-1}-\tilde{\varkappa }\right\|}_{\hbar } & \leq \left(1-\left[{℘}_{n-2}\left(1-\left(M_{\Psi }M_{z}+M\right)\right)-\left(M_{\Psi }M_{z}+M\right)\right]\right){\left\|\omega_{n-2}-\tilde{\varkappa }\right\|}_{\hbar }\\ & \;\vdots \\ {\left\|\omega_{1}-\tilde{\varkappa }\right\|}_{\hbar } & \leq \left(1-\left[{℘}_{0}\left(1-\left(M_{\Psi }M_{z}+M\right)\right)-\left(M_{\Psi }M_{z}+M\right)\right]\right){\left\|\omega_{0}-\tilde{\varkappa }\right\|}_{\hbar }, \end{array}$$

and inductively


$$\begin{array}{c} {\left\|\omega_{n+1}-\tilde{\varkappa }\right\|}_{\hbar }\leq {\left\|\omega_{0}-\tilde{\varkappa }\right\|}_{\hbar }\prod_{j=0}^{n}\left(1-\left[{℘}_{j}\left(1-\left(M_{\Psi }M_{z}+M\right)\right)-\left(M_{\Psi }M_{z}+M\right)\right]\right). \end{array}$$ (57)

Because $\rho_{j},\xi_{j},\lambda_{j},{℘}_{j}\in (0,1)$ and applying the hypotheses (A_6_), we get


$$\begin{array}{c} {℘}_{n}<1\text{ and }1-\left(M_{\Psi }M_{z}+M\right)<1. \end{array}$$

From the fact that $1-\omega \leq e^{-\omega },$ we have


$$\begin{array}{c} {\left\|\omega_{n+1}-\tilde{\varkappa }\right\|}_{\hbar }\leq \left\|\omega_{0}-\eta \right\|e^{-\sum_{j=0}^{\infty }\left[{℘}_{j}\left(1-\left(M_{\Psi }M_{z}+M\right)\right)-\left(M_{\Psi }M_{z}+M\right)\right]}. \end{array}$$

Passing n→∞, and using the assumption $\sum_{n=0}^{\infty }{℘}_{n}=\infty ,$ we have


$$\begin{array}{c} \lim_{n\to \infty }{\left\|\omega_{n}-\tilde{\varkappa }\right\|}_{\hbar }=0. \end{array}$$

This proves that the MZI (41) converges strongly to a unique solution $\tilde{\varkappa }.$

**Example 3.** *Consider the following integral equation:*


$$\begin{array}{c} \Upsilon \left(\zeta ,\delta \right)=\frac{\zeta \delta }{3}-\frac{\zeta^{3}\delta^{3}}{48}+\int_{0}^{\zeta }\int_{0}^{\delta }\frac{\zeta \delta }{4}\Upsilon \left(b,c\right)dbdc. \end{array}$$ (58)

*The unique solution to the problem (58) is* $\Upsilon \left(\zeta ,\delta \right)=\frac{\zeta \delta }{3}$*, as can be readily verified for all* ζ,δ∈[0,1]×[0,1]. *Let* z(Υ(ζ,δ))=Υ(ζ,δ) *for all* ζ,δ∈[0,1]×[0,1] *and* ζδ∈[0,1]. *Then, we have*


$$\begin{array}{c} \Psi \left(\zeta ,\delta ,z\left(\Upsilon \left(\zeta ,\delta \right)\right)\right)=\frac{\zeta \delta }{3}\left(1-\frac{\zeta^{2}\delta^{2}}{16}\right), \end{array}$$

*and*


$$\begin{array}{c} ℑ\left(\zeta ,\delta ,b,c,\Upsilon \left(b,c\right)\right)=\frac{\zeta \delta }{4}\Upsilon \left(b,c\right). \end{array}$$

*Clearly*


$$\begin{array}{c} \left|z\left(\Upsilon \left(\zeta ,\delta \right)\right)-z\left(\tilde{\varkappa }\left(\zeta ,\delta \right)\right)\right|=1.\left|\Upsilon \left(\zeta ,\delta \right)-\tilde{\varkappa }\left(\zeta ,\delta \right)\right|, \end{array}$$

*which leads to* $M_{z}=1.$ *Also,*


$$\begin{array}{cc} & \left|\Psi \left(\zeta ,\delta ,z\left(\Upsilon \left(\zeta ,\delta \right)\right)\right)-\Psi \left(\zeta ,\delta ,z\left(\tilde{\varkappa }\left(\zeta ,\delta \right)\right)\right)\right|\\ & \;=\left|\Upsilon \left(\zeta ,\delta \right)\left(1-\frac{\zeta^{2}\delta^{2}}{16}\right)-\tilde{\varkappa }\left(\zeta ,\delta \right)\left(1-\frac{\zeta^{2}\delta^{2}}{16}\right)\right|\\ & \;=\left|1-\frac{\zeta^{2}\delta^{2}}{16}\right|\left|\Upsilon \left(\zeta ,\delta \right)-\tilde{\varkappa }\left(\zeta ,\delta \right)\right|. \end{array}$$

*For the Lipschitz condition to be satisfied, the term* $\left(1-\frac{\zeta^{2}\delta^{2}}{16}\right)$ *must remain bounded. Its upper bound is achieved when* ζ=1=δ*, yielding* $\left(1-\frac{\zeta^{2}\delta^{2}}{16}\right)\leq \frac{15}{16}$*, for all* ζ,δ∈[0,1]*. Consequently,*


$$\begin{array}{c} \left|\Psi \left(\zeta ,\delta ,z\left(\Upsilon \left(\zeta ,\delta \right)\right)\right)-\Psi \left(\zeta ,\delta ,z\left(\tilde{\varkappa }\left(\zeta ,\delta \right)\right)\right)\right|\leq \frac{15}{16}\left|\Upsilon \left(\zeta ,\delta \right)-\tilde{\varkappa }\left(\zeta ,\delta \right)\right|, \end{array}$$

*which implies that* $M_{\Psi }=\frac{15}{16}.$ *Further,*


$$\begin{array}{c} \left|ℑ\left(\zeta ,\delta ,b,c,z\left(\Upsilon \left(\zeta ,\delta \right)\right)\right)-ℑ\left(\zeta ,\delta ,b,c,z\left(\tilde{\varkappa }\left(\zeta ,\delta \right)\right)\right)\right|\leq \frac{\zeta \delta }{4}\left|z\left(\Upsilon \left(\zeta ,\delta \right)\right)-z\left(\tilde{\varkappa }\left(\zeta ,\delta \right)\right)\right|, \end{array}$$

*hence* $M_{ℑ}\left(\zeta ,\delta ,b,c\right)=\frac{\zeta \delta }{4}.$ *Finally, to evaluate M, we have*


$$\begin{array}{cc} \int_{0}^{\zeta }\int_{0}^{\delta }\frac{\zeta \delta }{4}e^{\hbar \left(b+c\right)}dbdc & =\left(\frac{\zeta \delta }{4}\right)\int_{0}^{\zeta }\int_{0}^{\delta }e^{\hbar \left(b+c\right)}dbdc\\ & =\left(\frac{\zeta \delta }{4}\right)\int_{0}^{\zeta }\left(\frac{e^{\hbar \delta }-1}{\hbar }\right)e^{\hbar c}dc\\ & =\left(\frac{\zeta \delta }{4\hbar^{2}}\right)\left(e^{\hbar \left(\delta +\zeta \right)}-e^{\hbar \delta }-e^{\hbar \zeta }+1\right)\\ & \leq \left(\frac{1}{4\hbar^{2}}\right)\left(e^{\hbar \left(\delta +\zeta \right)}+1\right)\\ & =\left(\frac{1}{4\hbar^{2}}\right)\left(1+\frac{1}{e^{\hbar \left(\delta +\zeta \right)}}\right)e^{\hbar \left(\delta +\zeta \right)}\\ & \leq \left(\frac{2}{4\hbar^{2}}\right)e^{\hbar \left(\delta +\zeta \right)}=\left(\frac{1}{2\hbar^{2}}\right)e^{\hbar \left(\delta +\zeta \right)}. \end{array}$$

*Thus,* $M=\frac{1}{2\hbar^{2}}$ *and* $M_{\Psi }M_{z}+M=\left(\frac{15}{16}\times 1+\frac{1}{2\hbar^{2}}\right)=\frac{15}{16}+\frac{1}{2\hbar^{2}}<1,$ *this is true when* $\hbar >2\sqrt{2}>0.$ *Consequently, all the stipulations (A*_*1*_*)-(A*_*6*_*) are fulfilled. Therefore, the integral* equation (58) *admits a unique solution* $\Upsilon \left(\zeta ,\delta \right)=\frac{\zeta \delta }{3}$*, and its Picard sequence of successive approximations converges uniformly to the solution.*

## 7 Conclusion and open problems

Iterative schemes are paramount for reckoning FPs, especially when dealing with complex nonlinear integral equations, because direct analytical solutions are often intractable or nonexistent. By transforming a nonlinear integral equation into an equivalent FP problem, iterative methods generate a sequence of successive approximations that, under certain conditions, converge to the unique FP, which in turn represents the solution to the original integral equation. These schemes not only provide a practical means of approximating solutions to otherwise unsolvable problems arising in various scientific and engineering disciplines but also allow for the analysis of convergence rates and stability, facilitating the development of more efficient and robust numerical techniques.

So, in this paper, the existence of common FPs for C−α−NE mappings is primarily explored. A novel four-step iterative algorithm, termed the MZI, is introduced as the main contribution, specifically developed for pairs of mappings. This algorithm is then leveraged to derive several weak and strong convergence results, thereby demonstrating the existence of these common FPs. Constructive illustrative examples are provided to validate the theoretical results. Additionally, the practical applicability of the proposed algorithm is showcased by using it to approximate solutions to a certain class of nonlinear VFIEs in BSs, further supported by a relevant integral equation example.

Finally, the following points are designated for future research:

- We can find a common solution to the variational inequality problem by defining a nonlinear self-mapping ℜ in a Hilbert space Ξ (equipped with an inner product) and then applying our iteration (41). The problem is stated as follows: find $\omega^{*}\in \Xi$ such that

$$\begin{array}{c} \left\langle ℜ\omega^{*},\omega -\omega^{*}\right\rangle \geq 0,\;\forall \omega \in \Xi . \end{array}$$

- Variational inequalities serve as vital modeling instruments in diverse areas like engineering mechanics, transportation, economics, and mathematical programming [41,42].
- Generalizing our algorithm to gradient and extra-gradient projection methods is possible. Such methods are highly significant for identifying saddle points and resolving numerous optimization challenges [43].
- We can accelerate the convergence of our proposed algorithm by incorporating shrinking projection and CQ terms. These methods are known to significantly boost algorithm performance and achieve strong convergence [44,45].
- By defining the mapping ℜ as α−inverse strongly monotone and adding an inertial term to our algorithm, the inertial proximal point algorithm is obtained. This algorithm is highly applicable in diverse areas like monotone variational inequalities, image restoration problems, convex optimization problems, and split convex feasibility problems [46,47]. More precisely, these problems can be formulated as mathematical models for fields such as machine learning and linear inverse problems.
- Our algorithm can also be applied to second-order differential equations and fractional differential equations. These can be transformed into integral equations via Green’s function, making them straightforward to handle and solve using the same approach as in last part.
- The error of our present iteration can be quantified.

## Abbreviations

FP: fixed point
CAM: contraction mapping
BS: Banach space
NE: nonexpansive
MZI: modified Z−iteration
VFIE: Volterra–Fredholm integral equation
